# Fabrication of rigidity and space variable protein oligomers with two peptide linkers†
†Electronic supplementary information (ESI) available. See DOI: 10.1039/c9sc04158c


**DOI:** 10.1039/c9sc04158c

**Published:** 2019-10-07

**Authors:** Hyeokjune Choi, Hyoin Park, Kabi Son, Ho Min Kim, Yongwon Jung

**Affiliations:** a Department of Chemistry , KAIST , 291 Daehak-ro, Yuseong-gu , Daejeon 34143 , Republic of Korea . Email: ywjung@kaist.ac.kr ; Fax: +82-42-350-2810 ; Tel: +82-42-350-2817; b Graduate School of Medical Science & Engineering , KAIST , Daejeon 34141 , Republic of Korea; c Center for Biomolecular & Cellular Structure , Institute for Basic Science (IBS) , Daejeon 34126 , Republic of Korea

## Abstract

Supramolecular protein assemblies have garnered considerable interest due to their potential in diverse fields with unrivaled attainable functionalities and structural accuracy. Despite significant advances in protein assembly strategies, inserting long linkers with varied lengths and rigidity between assembling protein building blocks remains extremely difficult. Here we report a series of green fluorescent protein (GFP) oligomers, where protein building blocks were linked *via* two independent peptide strands. Assembling protein units for this two-peptide assembly were designed by flopped fusion of three self-assembling GFP fragments with two peptide linkers. Diverse flexible and rigid peptide linkers were successfully inserted into high-valent GFP oligomers. In addition, oligomers with one flexible linker and one rigid linker could also be fabricated, allowing more versatile linker rigidity control. Linker length could be varied from 10 amino acids (aa) even up to 76 aa, which is the longest among reported protein assembling peptide linkers. Discrete GFP oligomers containing diverse linkers with valencies between monomers to decamers were monodispersely purified by gel elution. Furthermore, various functional proteins could be multivalently fused to the present GFP oligomers. Binding assays, size exclusion chromatography, dynamic light scattering, circular dichroism, differential scanning calorimetry, and transmission electron microscopy suggested circular geometries of the GFP oligomers and showed distinct characteristics of GFP oligomers with length/rigidity varied linkers. Lastly, a surface binding study indicated that more spaced oligomeric binding modules offered more effective multivalent interactions than less spaced modules.

## Abstract 2

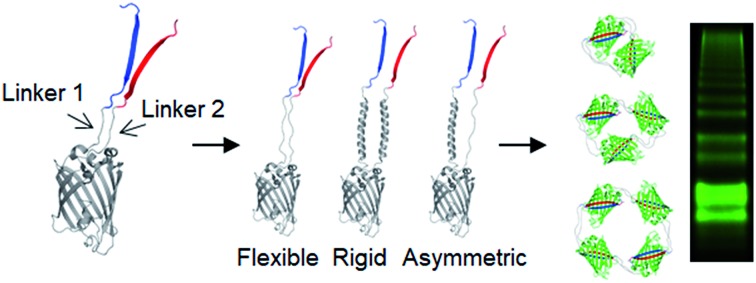
A new two peptide-linked protein assembly strategy was developed for diverse linker length and rigidity variation on protein oligomers.

## Introduction

Biological systems utilize precisely organized assemblies of diverse protein building blocks. Many artificial protein assemblies have been investigated not only to understand these natural assembly processes but also to create novel supramolecular protein architectures, which can offer unmatched functionalities and atomic level structural accuracy.^1^,^2^ In many cases, protein units are repeatedly linked to polymeric (or oligomeric) assembly forms *via* specific and often rigid interactions. These interactions include those between metal–ligand,^3^–^5^ protein–ligand (co-factor),^6^–^8^ and protein–peptide (protein).^9^–^13^ While these protein polymers have been assembled mostly with heterogeneous populations, discrete supramolecular protein (mostly cage) structures have also been computationally designed and fabricated by symmetric assemblies of multimeric protein subunits with atomic accuracy.^14^–^17^ Discrete protein oligomers with defined valency were also prepared by multimer isolation from protein polymer mixtures.^12^

Despite these rapidly developing protein assembly strategies, varying the length and rigidity of linkers between assembled protein units is still highly challenging. Most symmetric protein assembly approaches by computational design require short and rigid linkers between binding protein domains or even direct protein contact *via* designed interfaces.^2^,^14^ For protein polymers, incorporating relatively long (both peptide and synthetic chemical) linkers between monomeric building blocks leads to increased intramolecular assembly, which produces dominantly low-valent oligomers (Table S1†). Multiple studies have demonstrated the importance of spacing between assembled functional proteins. Optimization of the distance between multiple protein binding domains is critical to maximize binding affinities to multimeric target proteins *via* multivalent interactions.^18^,^19^ Spacing between multiple ligands against cell surface receptors is also one of the key regulation factors for cellular receptor signaling.^20^,^21^ At present, however, spacing between assembled proteins is mostly varied by adapting DNA scaffolds, which are synthetically fused to protein units. Developing new assembly strategies that allow variations on linkers between assembled proteins will greatly diversify the structures and functions of supramolecular protein assemblies.

**Scheme 1:**
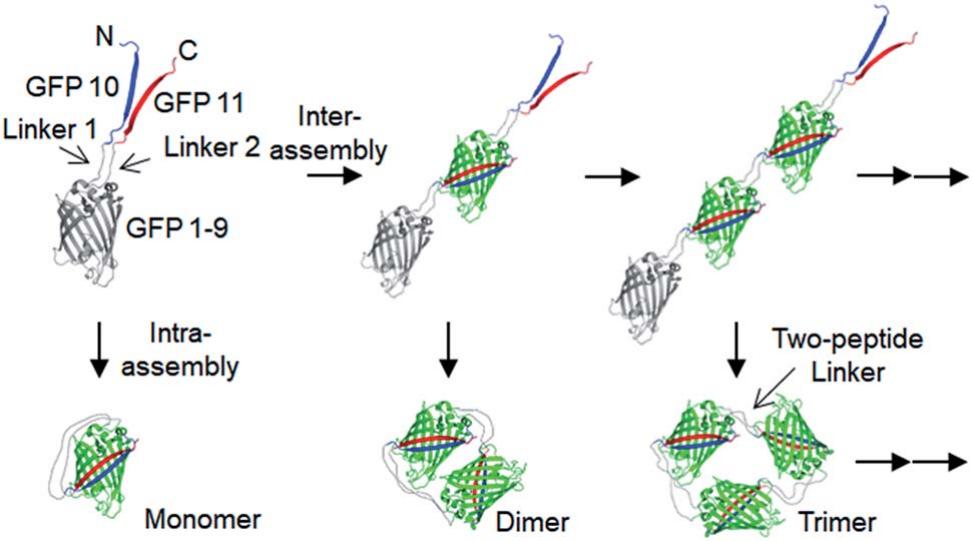
Schematic representation of expected oligomerization processes of two peptide-linked GFP oligomers. Blue, red, and gray denote GFP 10, GFP 11, and GFP 1–9, respectively. The N- and C-termini of the monomeric building block are depicted as ‘N’ and ‘C’, respectively.

Here, we developed a new in-cell protein assembly approach, where protein units (GFP) were linked *via* two peptide strand linkers with varied length and flexibility. The tripartite split GFP fragments (GFP 1–9, GFP 10 and GFP 11),^22^ which can self-assembled to form mature GFP, were utilized for this two-peptide linker assembly. Flopped fusion of three GFP fragments provided a protein building block with an ideal assembling orientation for a high-degree of intermolecular oligomerization *via* two peptide linkers (Scheme 1). Importantly, linkers with various lengths and flexibility could be inserted, while maintaining effective GFP oligomerization. By careful control of linker flexibility, we were able to fabricate highly spaced and flexible GFP oligomers, where the longest two-peptide linkers consisted of one 38-amino acids (aa) flexible and one 76-aa rigid peptide linkers. Various functional proteins were successfully displayed on these GFP oligomers, and discrete oligomers could be purified by gel electro-elution. The exact valence numbers of discrete GFP oligomers were confirmed by functional binding assays. We also assessed the spatial arrangements of oligomers by visualizing through transmission electron microscopy (TEM), and demonstrated how the linker rigidity affects the elution profiles of GFP oligomers during size-exclusion chromatography (SEC). Dynamic light scattering (DLS), circular dichroism (CD), and differential scanning calorimetry (DSC) analyses were also conducted to investigate various structural features of GFP oligomers with different linkers. Furthermore, we examined binding characteristics of binding domain-fused GFP oligomers with varied linkers using surface plasmon resonance (SRP) analysis.

## Results and discussion

### Design and cellular assembly of spaced GFP oligomers *via* two peptide linkers

Spontaneous self-assembly of two split superfolder GFP fragments offers a unique protein-peptide interaction, which is weak but becomes extremely stable once mature GFP with 11 beta strands is formed.^23^ GFP is also an attractive building block for protein assemblies with inherent fluorescent signals and high solubility. Previously, we demonstrated that strand-swapped GFP 11-GFP 1–10 fusion proteins (GFP 1–10 for the first 10 strands and GFP 11 for the last strand) can be assembled into polygonal GFP oligomers with various sizes in *E. coli*.^12^ However, like many other protein linking approaches, increasing the length of the single peptide linker (even to 6 aa) between GFP units severely prevented protein oligomerization. Another distinct self-assembling split-GFP system with three fragments (GFP 1–9, GFP 10, and GFP 11) was also reported.^22^ When GFP 10 and GFP 11 strands were brought together in close proximity, these two strands can self-assemble with GFP 1–9, eventually forming a mature GFP protein with bright fluorescence (Fig. S1†). We designed a new self-assembling building block, which contains N-terminal GFP 10 and C-terminal GFP 11 fusions on GFP 1–9 *via* two peptide linkers: GFP 10-linker 1-GFP (1–9)-linker 2-GFP 11 (Scheme 1). We envisioned that this unit would offer an ideal assembling orientation for high-valent GFP oligomerization and might allow wide variations on linker length and rigidity with its unique two-peptide linking strands.

**Fig. 1:**
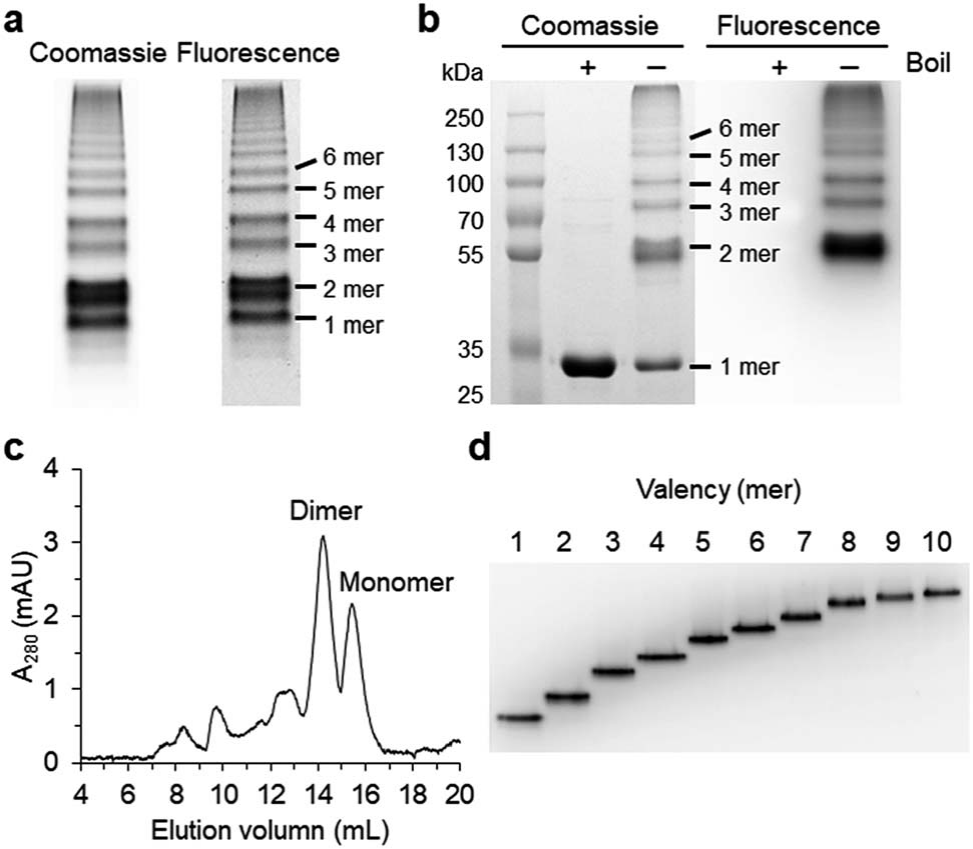
Cellular assembly of GFP oligomers with two peptide linkers. (a) Native PAGE analysis of GFP oligomers (10aa/10aa). The gel was analyzed by fluorescent analyzer (right) and Coomassie blue staining (left). (b) SDS-PAGE analysis of GFP oligomers with or without boiling. Estimated oligomeric states were indicated. (c) Size-exclusion chromatography (SEC) of GFP oligomers on a superdex 200 column (10/300 GL). (d) Native PAGE analysis of gel-purified GFP oligomers from monomer to decamer.

Flexible 10 aa peptides were first introduced as two linkers (linker 1: GTDVGSGGGS and linker 2: GGGSGGGSTS; 10aa/10aa) (Table 1). When the resulting protein building block is overexpressed in *E. coli*, a wide range of fluorescent GFP oligomers from monomer to over decamer were effectively assembled, as demonstrated by native polyacrylamide gel electrophoresis (PAGE) (Fig. 1a). GFP oligomers were produced in an over 100 mg scale from 1 L of *E. coli* flask culture and easily purified using Ni-affinity chromatography. Oligomers were stable during multi-step purification processes such as sonication, centrifugation, and affinity column purification without any noticeable loss of fluorescence (Fig. S2†). More precise GFP oligomer valency was estimated by gel analysis in the presence of a 0.1% denaturing detergent, sodium dodecyl sulfate (SDS), for size-dependent separation. The oligomers were mostly stable under 0.1% SDS, showing discrete fluorescent bands that correspond to multiple GFP units, whereas only a monomer band was exhibited in boiled samples (Fig. 1b). While GFP oligomers were not discretely separated by SEC, SEC and native gel analysis with fluorescent signal quantitation indicate that close to 43% of expressed (10aa/10aa) GFP building blocks were polymerized in cells into high molecular weight oligomers (≥3 mer) (Fig. 1c and S3†).

**Table 1:** Sequences and lengths of peptide linkers of GFP oligomers

GFP oligomers	Linker (linker 1: L1, linker 2: L2)	Amino acids
4aa/4aa^*a*^	L1: GGGS	4/4
	L2: GSTS	
10aa/10aa	L1: GTDVGSGGGS	10/10
	L2: GGGSGGGSTS	
15aa/15aa	L1: GTDVGSGGGSGGGGS	15/15
	L2: GGSGGGGGSGGGSTS	
22aa/22aa^*a*^	L1: GTDVGSGGGSGGGGSGGGSEGS	22/22
	L2: GGSGGGGGSGGGSTSGGGSEGS	
30aa/30aa^*a*^	L1: GTDVGSGGGSGGGGSGGGSEGSGGSGGGSG	30/30
	L2: GGSGGGGGSGGGSTSGGGSEGSGGSGGGSG	
EK_*n*_/EK_*n*_	L1: GSGS(EAAAK)_*n*_GSGS (*n* = 2, 3, 4, 5, 6)	5n+8/5n+8
	L2: GSGS(EAAAK)_*n*_GSGS (*n* = 2, 3, 4, 5, 6)	
EP_*n*_/KP_*n*_	L1: GSGS(EP)_*n*_GSGS (*n* = 4, 6^*a*^)	2n+8/2n+8
	L2: GSGS(KP)_*n*_GSGS (*n* = 4, 6)	
(H4)_2_/(H4)_2_	L1: GSGS(EAAAK)_4_ALEA(EAAAK)_4_GSGS	52/52
	L2: GSGS(EAAAK)_4_ALEA(EAAAK)_4_GSGS	
(H4)_3_/(H4)_3_	L1: GSGS(EAAAK)_4_ALEA(EAAAK)_4_GSGS(EAAAK)_4_GSGS	76/76
	L2: GSGS(EAAAK)_4_ALEA(EAAAK)_4_GSGS(EAAAK)_4_GSGS	
(H4)_2_/30aa	L1: GSGS(EAAAK)_4_ALEA(EAAAK)_4_GSGS	52/30
	L2: GGSGGGGGSGGGSTSGGGSEGSGGSGGGSG	
(H4)_3_/38aa	L1: GSGS(EAAAK)_4_ALEA(EAAAK)_4_GSGS(EAAAK)_4_GSGS	76/38
	L2: GGSGGGGGSGGGSTSGGGSEGSGGSGGGSGGGGSGGGS	
(EP)_6_/22aa	L1: GSGS(EP)_6_GSGS	20/22
	L2: GGSGGGGGSGGGSTSGGGSEGS	

^*a*^GFP oligomers 4aa/4aa, 22aa/22aa, 30aa/30aa, and EP_6_/EP_6_ were only marginally oligomerized.

Monodisperse oligomers were next isolated in a size-dependent manner from monomer to decamer by gel electro-elution (Fig. 1d).^12^ Assembled and gel-purified GFP oligomers were intact during multistep gel elution processes. In addition, oligomers from monomer to hexamer all showed identical fluorescence spectra, indicating stable GFP maturation regardless of protein valency (Fig. S4†). This data also suggests that purified GFP oligomers were all intra-molecularly circularized forms without any free GFP 1–9 and GFP 10/GFP 11strands (Scheme 1). To further support circularization of GFP oligomers, free GFP 1–9 was added to purified oligomers as well as to GFP with free GFP 10/GFP 11 strands as a control. GFP 1–9 was assembled with free GFP 10/GFP 11 strands to form mature GFP but not with GFP oligomers, indicating the absence of free GFP 10/GFP 11 strands on GFP oligomers due to complete circularization (Fig. S5†). Eluted GFP oligomers can be concentrated over 0.3 mg mL^–1^ and were stable in phosphate-buffered saline (PBS) at 4 °C for at least 6 months. For GFP trimer and tetramer, 2–4 mg of each oligomer could be prepared from 1 L of *E. coli* culture, and 1–2 mg of GFP oligomers from 5 mer to 10 mer could be homogenously isolated from the same protein mixture.

### Rigidity and length variation of two peptide linkers

**Fig. 2:**
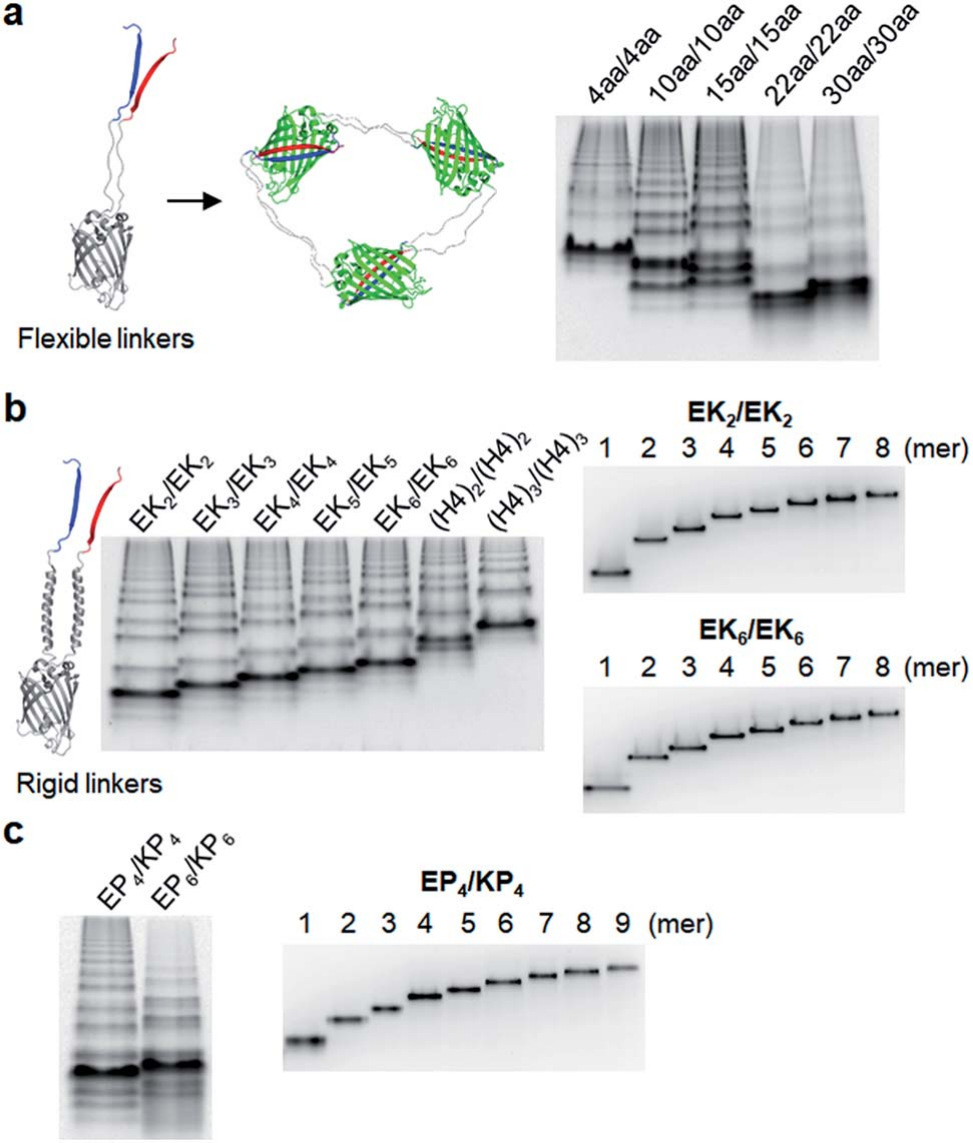
Rigidity and length variation of two peptide linkers on GFP oligomers. (a) Native PAGE analysis of GFP oligomers with two flexible linkers. Linker lengths are varied from 4 aa to 30 aa. (b) Native PAGE analyses of GFP oligomer mixtures with length-varied rigid helical linkers (left) and purified GFP oligomers (EK_2_/EK_2_ and EK_6_/EK_6_; right). (c) Native PAGE analysis of GFP oligomers with proline-rich linkers (left) and purified GFP oligomers (EP_4_/KP_4_; right).

We next inserted a wide range of two peptide linkers with different lengths and rigidity on GFP oligomers. Flexible linkers are usually composed of small or hydrophilic amino acids such as glycine, serine, and threonine to render both solubility and flexibility, while rigid linkers generally include helical sequences or prolines that act as a structural disruptor.^24^ The exemplary linkers widely used in protein fusion are (GGGGS)_*n*_, (GS)_*n*_, and (G)_*n*_ for flexible linkers and (EAAAK)_*n*_, (EP)_*n*_, (KP)_*n*_, (AP)_*n*_, and (TPR)_*n*_ for rigid linkers. From the initial 10 aa linkers (Fig. 1), we first adjusted the length of the flexible linkers, which mostly are composed of glycines and serines (Table 1). Shortening each of two linkers to 4 aa (4aa/4aa) led to significantly reduced formation of high-valent oligomers (Fig. 2a and S6†), possibly due to steric hindrance between GFP subunits. On the other hand, GFP oligomers with 15 aa linkers (15aa/15aa) were assembled in a similar manner to 10aa/10aa oligomers. Longer flexible linkers (22 aa and 30 aa), however, clearly prevented high-valent oligomer assemblies, yielding mostly intra-molecularly assembled monomers.

Next, we introduced rigid peptide linkers such as helical EAAAK repeats (EK_*n*_) and proline rich EP/KP repeats (EP_*n*_/KP_*n*_).^25^,^26^ Identical helical peptides containing from 2 (EK_2_) to 6 (EK_6_) sequentially increased EAAAK repeats were inserted between GFP 1–9 and GFP10/11 strands (Table 1). In addition, much longer ((H4)_2_: 52 aa and (H4)_3_: 76 aa) but less stiff peptides,^27^ which contain 8 and 12 EAAAK repeats, respectively, were also introduced. Interestingly, these GFP building blocks with helical linkers were all successfully assembled into a series of high-valent GFP oligomers (Fig. 2b), unlike those with long fully flexible linkers. In addition, these oligomers were stably and homogeneously isolated again by gel-elution (Fig. 2b). Rigid linkers might prevent prompt intra-protein assemblies and allow more inter-protein assemblies to produce diverse GFP oligomers with similar valency distributions regardless of linker lengths. These GFP oligomers offer the first multivalent protein scaffolds, where backbone protein units were linked *via* two helical peptide linkers with incrementally variable lengths (from 18 aa to 76 aa). We also inserted short proline rich EP and KP repeats between ‘GFP 10 and GFP 1–9’ and ‘GFP 1–9 and GFP 11’, respectively. EP and KP repeats were observed in a natural stiff peptide,^25^ and here, both sequences were used together as two peptide linkers to avoid repulsion between two charged linkers. GFP oligomers bearing four repeats of EP and KP (EP_4_/KP_4_) were well constructed and purified as stable monodisperse forms, whereas the subunits with six repeats (EP_6_/KP_6_) were poorly oligomerized, presumably owing to high structural constraints on constructs (Fig. 2c).

### Insertion of asymmetric two peptide linkers

**Fig. 3:**
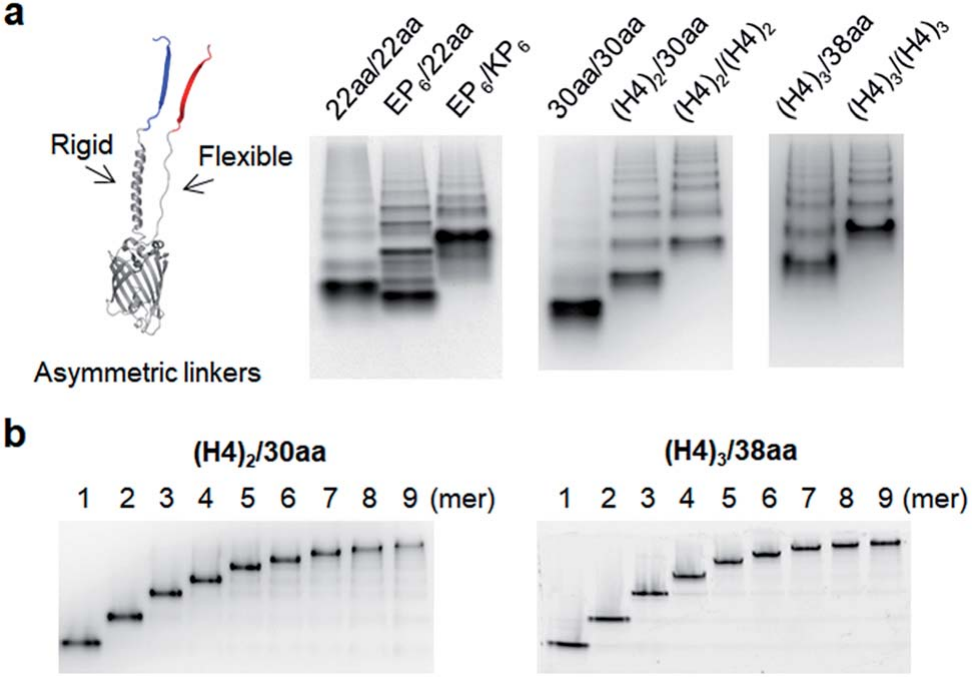
GFP oligomers with asymmetric two peptide linkers. (a) Native PAGE analyses of GFP oligomers with various flexible/rigid asymmetric peptide linkers. (b) Native PAGE analyses of purified GFP oligomers: (H4)_2_/30aa (left) and (H4)_3_/38aa (right) from monomers to nonamers.

Although highly spaced GFP oligomers were fabricated with two helical peptide linkers, GFP oligomerization *via* long flexible linkers was largely inefficient. To vary the rigidity of peptide linkers between these diversely separated GFP units of GFP oligomers, we utilized two peptide linkers with different rigidity: rigid linker 1 between GFP 10 and GFP 1–9; flexible linker 2 between GFP 1–9 and GFP 11 (Fig. 3). First, the highly rigid EP_6_ peptide (20 aa) was combined with the flexible 22 aa peptide (Table 1). Interestingly, high-valent GFP oligomers were more effectively formed with asymmetric EP_6_/22aa linkers than fully flexible 22aa/22aa or rigid EP_6_/KP_6_ linkers (Fig. 3a). Reversing two peptide linkers (flexible linker 1 and rigid linker 2 for 22aa/EP_6_) did not affect the oligomerization degree or protein expression yield (Fig. S7†). Next, the longer 30 aa flexible peptide was combined with the helical (H4)_2_ peptide, which is 52 aa but can have a similar length to the extended 30 aa flexible peptide with its helical structure. GFP units with these asymmetric linkers ((H4)_2_/30aa) were also successfully oligomerized.

To further increase the spacing of asymmetric GFP oligomers, linker 2 of the most extended (H4)_3_/(H4)_3_ GFP oligomers was replaced with a long 38 aa flexible peptide. The resulting (H4)_3_/38aa also showed a clear ladder form of oligomers on a native gel (Fig. 3a). The present two-peptide linker protein assembly allowed the insertion of extremely long peptide linkers between protein building blocks while also varying the rigidity of these linkers. To our knowledge, (H4)_3_/(H4)_3_ and (H4)_3_/38aa GFP oligomers represent the longest peptide linkers connecting monomeric protein subunits (Table S1†). Discrete GFP oligomers with long and asymmetric rigid/flexible peptide linkers were also stably and monodispersely isolated from the mixtures (Fig. 3b). We even further extended linkers to (H4)_4_/50aa. While (H4)_4_/50aa GFP oligomers showed a moderate oligomerization level, oligomers were not separated well on a native gel due to their large sizes (Fig. S8†), limiting the use of (H4)_4_/50aa as multivalent protein scaffolds. Additionally, we applied our assembly approach to other GFP color variants to examine how slight variation on the protein structure affects the present protein assembly and also if FP oligomers with different colors can be generated. Upon single residue mutations, yellow FP oligomers were successfully formed with both short and long linkers, while oligomeric assembly was clearly less efficient for cyan and blue proteins (Fig. S9†).

### Multivalent fusion of functional proteins on GFP oligomers and valency determination

**Fig. 4:**
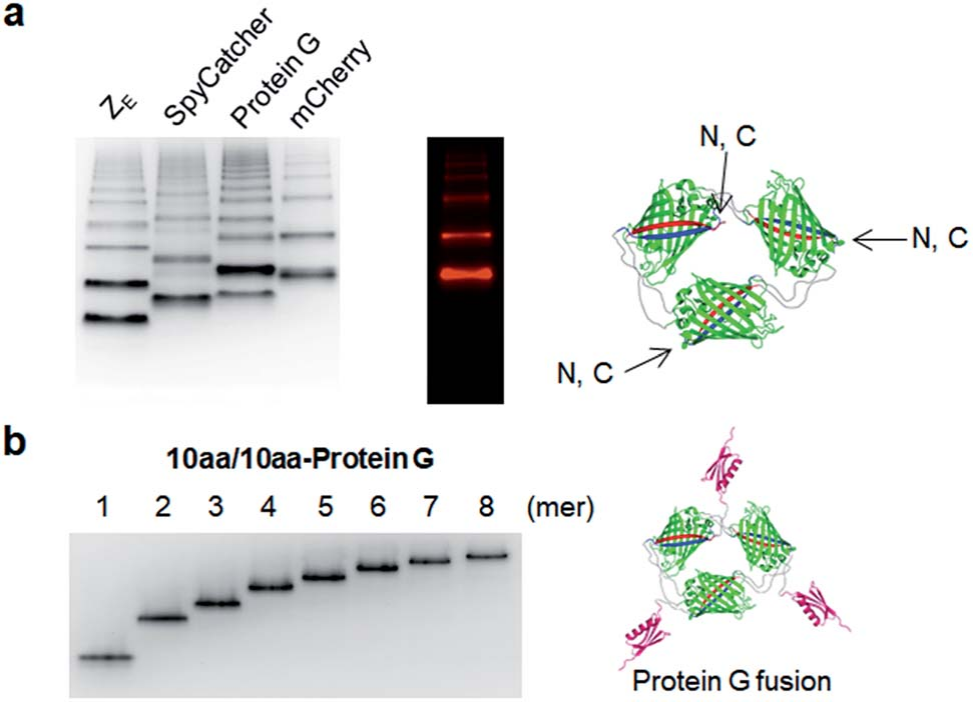
Multivalent functional protein display on GFP oligomers. (a) Native PAGE analysis of Z_E_, SpyCatcher, Protein G, and mCherry fused GFP oligomers (10aa/10aa). Red fluorescent signals on gels were additionally imaged for mCherry. Potential N- and C-termini of GFP building blocks on GFP trimer are indicated. (b) Native PAGE analysis of purified Protein G fused GFP oligomers (10aa/10aa) from monomer to octamer.

We next examined whether GFP oligomers with two peptide linkers can be used as protein scaffolds for multivalent functional protein display. Oligomer functionalization was conducted by simple genetic fusion of diverse protein/peptide ligands to monomeric GFP units. Both the N- and C-termini of the monomer are located on the edges of GFP building blocks, and thereby terminal fused ligands will likely face the outside of the GFP oligomer rings (Fig. 4a). We introduced diverse functional peptides or proteins such as a leucine zipper peptide (Z_E_),^28^ a glue protein (SpyCatcher),^29^ an antibody binding protein (Protein G)^30^ and red fluorescent protein mCherry^31^ at the C termini. All resulting protein units were successfully expressed and assembled to fluorescent GFP oligomers with diverse multivalency (Fig. 4a). GFP oligomers with the highly negative charged Z_E_ peptide (–7 net charge) showed large separations between oligomers during gel electrophoresis. mCherry-fused GFP oligomers exhibited both green and red fluorescence signals, indicating that fused mCherry retained its functionality. In addition, functionalized oligomers could also be discretely and stably purified by gel electro-elution, as demonstrated with protein G-fused oligomers (Fig. 4b).

**Fig. 5:**
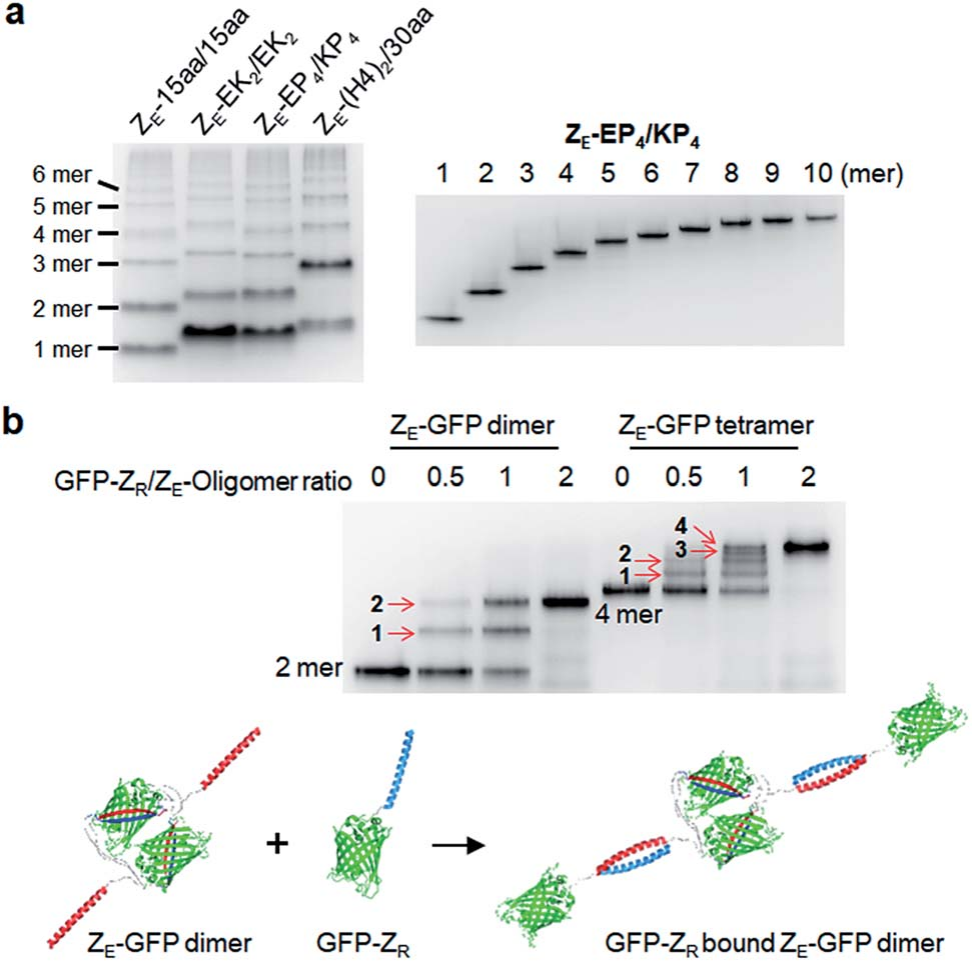
Valency confirmation of GFP oligomers by leucine zipper interaction. (a) Native PAGE analyses of Z_E_ fused GFP oligomers with various (flexible and rigid) two peptide linkers (left) and purified Z_E_-EP_4_/KP_4_ from monomer to decamer (right). (b) Electrophoretic mobility shift assays (EMSA) of Z_E_-(H4)_2_/30 aa GFP dimer and tetramer binding to GFP-Z_R_. Oligomers (1 μM Z_E_ concentration) were incubated with GFP-Z_R_ (0.5, 1, and 2 μM). Numbers of bound GFP-Z_R_ on Z_E_-GFP dimer and tetramer are indicated on corresponding protein complex bands with red arrows. Schematics of complex formation between GFP-Z_R_ and Z_E_-GFP dimer by high-affinity leucine zipper peptide (Z_E_/Z_R_) interaction is described below.

We utilized ligand-fused GFP oligomers to functionally confirm the accurate oligomer valencies (the numbers of GFP units), which were initially estimated based on oligomer sizes, as discussed in Fig. 1. To exploit rapid and stable interaction between leucine zipper Z_E_ and Z_R_ peptides (sequences in ESI†),^28^ the Z_E_ peptide was N-terminally fused to various GFP oligomers with different linker length and rigidity such as 15aa/15aa, EK_2_/EK_2_, EP_4_/KP_4_, and (H4)_2_/30aa. All Z_E_ fused proteins were well assembled to multivalent GFP oligomers, and each oligomer was homogeneously isolated (Fig. 5a and S10†). Precise Z_R_ binding valencies of Z_E_-fused GFP oligomers were functionally examined by native gel band-shift analyses with Z_R_ peptide-fused GFP (at the N-terminal) (GFP-Z_R_) (Fig. 5b). Z_E_-fused GFP oligomers were mixed with GFP-Z_R_ at varying concentration ratios ranging from 0.5 to 2 (GFP-Z_R_/Z_E_-GFP oligomer, Z_R_/Z_E_ concentration) for 1 h at 25 °C before gel analyses. Z_E_-GFP oligomer complexes with different numbers of bound GFP-Z_R_ were clearly discriminated (Fig. 5b). Expected Z_R_ binding valencies were clearly identified for all isolated oligomers with different two peptide linkers, as demonstrated with Z_E_-GFP dimers and tetramers (Fig. 5b and S11†). When two-fold excess of GFP-Z_R_ was added, all fused Z_E_ peptides on tested GFP oligomers were bound with GFP-Z_R_, indicating that all fused Z_E_ peptides are functional and ideally oriented for Z_R_ interaction on the present GFP oligomers with two peptide linkers.

### Structural characterization of two peptide-linked GFP oligomers

**Fig. 6:**
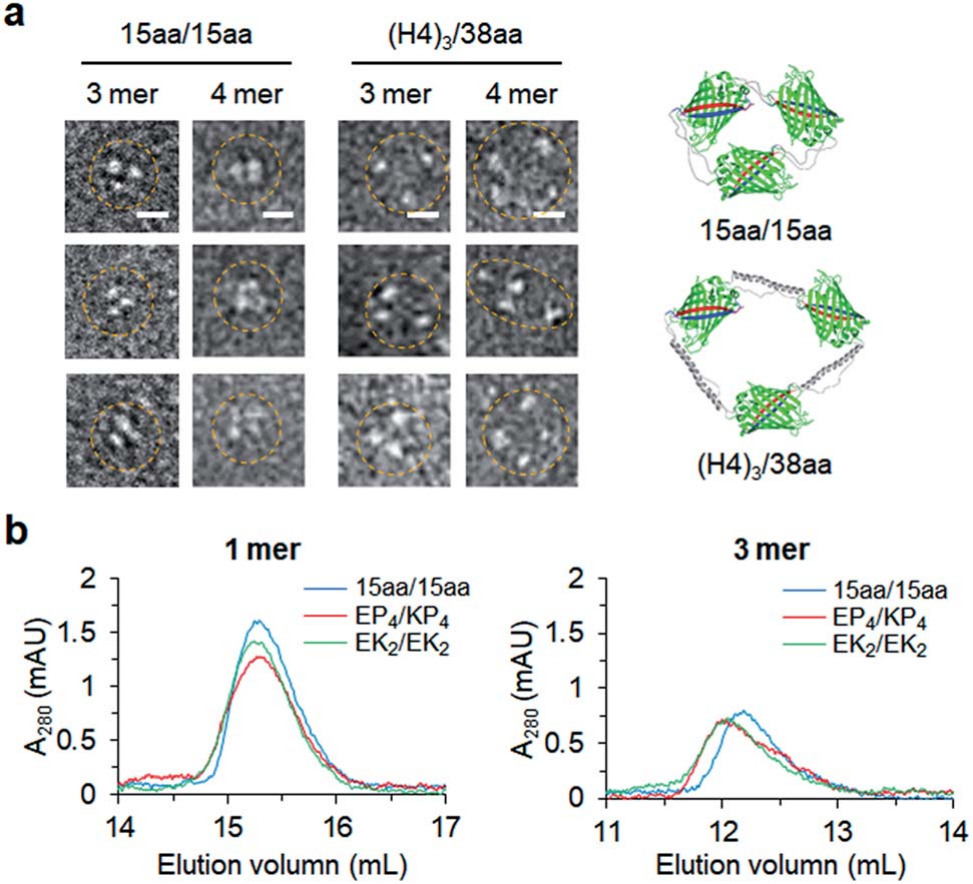
Structural studies of GFP oligomers. (a) Representative TEM images of 15aa/15aa and (H4)_3_/38aa GFP oligomers (trimers and tetramers). Scale bars, 10 nm. (b) Size-exclusion chromatography analyses of Z_E_ fused GFP oligomers (monomers and trimers).

Constant fluorescence intensities per an equal protein weight (Fig. S4†) and the lack of free GFP 10/11 strands (Fig. S5†) of assembled GFP oligomers suggested circularized oligomer structures. To further investigate protein geometries of two peptide-linked GFP oligomers, oligomers were analyzed with a transmitted electron microscopy (TEM) with negative staining. While many tightly and rigidly packed protein oligomers were directly imaged by TEM,^12^,^14^,^15^ visualization of widely spaced protein assemblies is highly challenging. Spaced GFP oligomers with flexible linkers (15aa/15aa) and the longest asymmetric linkers ((H4)_3_/38aa) were investigated to examine the effects of various long linkers on assembly geometries between GFP protein units. As expected, negative stained protein images of spaced GFP oligomers were highly heterogeneous, and it was difficult to identify dominant protein orientations (Fig. S12 and S13†). More spaced (H4)_3_/38aa oligomers showed even higher heterogeneity. Only a few representative images, in which all protein building blocks with expected oligomeric states were located, could be selected from many TEM images (Fig. 6a). Even for these images, spacing between GFP proteins was highly varied, where distances between protein building blocks were 7–18 nm for (H4)_3_/38aa and 4–7 nm for 15aa/15aa. Advancement on structural analysis methods will be crucial for direct visualization of highly spaced and flexible protein assemblies.

Structural characteristics of GFP oligomers were also investigated with size-exclusion chromatography (SEC), which is widely used for separation of proteins by their size and shape. For typical proteins, larger proteins are eluted (or migrate) faster than smaller proteins. To examine different chromatographic migration properties of GFP oligomers with different linkers, three GFP oligomers with similar sizes but with different linker rigidities (Z_E_-fused 15aa/15aa, EK_2_/EK_2_, and EP_4_/KP_4_) were subjected to SEC. Z_E_-fused oligomers were used because they are functionally further defined (Fig. 5 and S11†). GFP building blocks of these oligomers have similar molecular weights (35.8, 36.6, and 36.7 kDa, respectively). Indeed, monomeric assemblies were all eluted at a similar time (Fig. 6b). Monomers are intra-molecularly assembled with only one subunit, and thereby peptide linkers will have minimal effects on protein shapes (Scheme 1). On the other hand, a trimeric assembly with flexible linkers (15aa/15aa) was clearly eluted later than those with more rigid linkers (EK_2_/EK_2_ and EP_4_/KP_4_) (Fig. 6b). While their assembly geometries are highly heterogeneous (Fig. 6a), oligomers with rigid linkers might have higher populations of more widely spaced and stiff GFP oligomers (therefore acting as bigger proteins) than oligomers with flexible linkers. It is possible that long flexible peptide linkers were in more folded forms rather than in fully extended forms during SEC.

Relative size distributions of discrete GFP oligomers were also measured by dynamic light scattering (DLS). Monomers to tetramers of GFP oligomers with short (15aa/15aa) and long ((H4)_3_/38aa) linkers were examined. Oligomer sizes clearly increased as the valency increased, and sizes of (H4)_3_/38aa oligomers were larger than those of 15aa/15aa oligomers (Fig. S14†). In addition, size variations were generally higher for more spaced (H4)_3_/38aa oligomers than 15aa/15aa oligomers. We also examined structural heat stability of GFP oligomers with differential scanning calorimetry (DSC) and a heat titration test. DSC data indicated two melting temperatures of GFP oligomers (Fig. S15†). We believe that the first melting event (∼60 °C) corresponds to oligomer disassembly into monomeric units, and the second one (∼67 °C) to complete denaturation of these GFP units. Fluorescence loss after heating oligomers at ∼60 °C might also indicate oligomer disassembly at the temperature (Fig. S16†). Secondary structures of GFP oligomers were analyzed by circular dichroism (CD), which showed dominant beta-sheet signals for both 15aa/15aa and (H4)_3_/38aa oligomers but strong alpha-helix signals only for (H4)_3_/38aa oligomers with their excessive helical linker (Fig. S17†).

### Surface binding study of protein G functionalized GFP oligomers

**Fig. 7:**
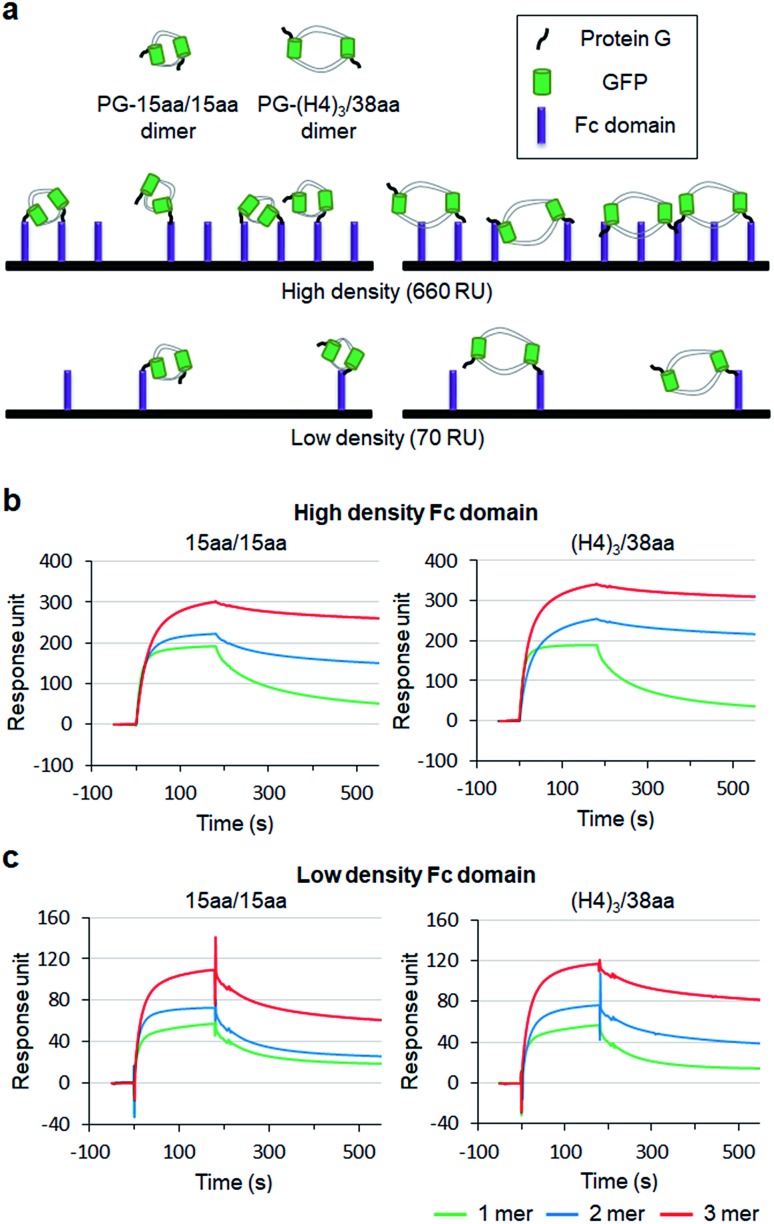
Multivalent surface interactions of protein G (PG) fused GFP oligomers. (a) Schematic representation of the binding between surface bound human Fc domains and PG-GFP dimers with different linkers (15aa/15aa and (H4)_3_/38aa). SPR sensorgrams of PG-15aa/15aa and PG-(H4)_3_/38aa binding to (b) the high density Fc domain surface (660 RU) and to (c) the low density Fc domain (70 RU). PG-GFP 1 mer, 2 mer, and 3 mer are marked in green, blue, and red, respectively.

To examine how biological behaviors of protein oligomers are influenced by inserted linkers, we conducted SPR-based multivalent protein binding analysis for GFP oligomers with short and long two peptide linkers. Protein G (PG), which binds to the antibody Fc domain, was fused to less spaced 15aa/15aa and more spaced (H4)_3_/38aa GFP oligomers. PG-fused GFP oligomers (monomers, dimers, and trimers) were discretely prepared and treated to Fc domain-covered chip surfaces (Fig. 7a). The Fc domain protein was immobilized onto SPR flat gold chip surfaces with relatively high (660 response unit, RU) and low (70 RU) protein surface densities. The same PG-GFP monomer concentration (100 nM) was used for all constructs, and therefore, dimer and trimer concentrations were 50 nM and 33 nM, respectively.

On the high density Fc domain surface, binding stability of PG-GFP proteins was clearly enhanced by increased valency, showing significantly slowed dissociation, likely by multivalent binding (Fig. 7b). Regardless of linkers, PG-GFP monomers similarly bind to this Fc domain surface. On the other hand, enhanced binding stability was noticeably greater for GFP oligomers with the longer (H4)_3_/38aa linkers than those with shorter 15aa/15aa (Fig. S18†). The data indicate that more spaced (H4)_3_/38aa PG-GFP oligomers have a higher chance to offer multivalent interactions since they can cover wider areas with more Fc domains. Since Fc domains are randomly distributed on a surface, spaced PG oligomers might also have a higher chance to find an ideal combination of Fc domain proteins for more effective (stronger) multivalent interactions.

When the surface Fc domain coverage on flat gold surfaces was lowered (70 RU), slowed dissociation by increased valency of GFP oligomers was mostly vanished (Fig. 7c). It is possible that many surface Fc domains are too far apart for oligomers to capture them simultaneously for multivalent binding. Still, overall binding signals increased as the valency of GFP oligomers increased, even though an assembly concentration decreased from monomer (100 nM) to dimer (50 nM) and to trimer (33 nM). We suspect that multimer-monomer binding might be stronger than monomer–monomer binding. The present SPR study indicates that proper spacing between binding modules is important for effective multivalent interactions against multiple surface binding targets (*e.g.* cell surface receptors).

## Conclusions

We developed a new two-peptide linked protein assembly strategy by designing protein building blocks with three split/self-assembling GFP fragments. Two peptide linkers allowed the insertion of various flexible or rigid long peptide sequences between GFP units. In particular, helical peptide linkers could be varied from 18 aa to impressive 76 aa, the longest linker reported thus far. Furthermore, the flexibility of helical linkers could also be varied by replacing one of two helical peptides with flexible peptides, leading to fabrication of highly spaced and rigidity variable GFP oligomers. Diverse functional proteins were multi-valently displayed on GFP oligomers, and these functional oligomers were discretely isolated with valencies ranging from monomer up to decamer. While overall production yields of GFP oligomers and rather complicated preparation processes must be improved, GFP oligomers will be valuable scaffolds for investigation of multivalent protein interactions with variable valency, spacing, and rigidity. Here, we demonstrated that more spaced binding oligomers offered more effective and strong multivalent interactions on surfaces. The present spaced GFP oligomers can also be used as valency and spacing variable protein building blocks for additional high-order protein assembly. However, one of the critical limitations when fabricating and exploiting flexible spaced protein assemblies is difficult direct structural confirmation, as also demonstrated in the present TEM study. Future work must also focus on developing new or combinatorial approaches to verify structures of flexible supramolecular protein architectures.

## Conflicts of interest

There are no conflicts to declare.

## Supplementary Material

Supplementary information
