# Supplementary material for: Fabrication of rigidity and space variable protein oligomers with two peptide linkers
Source: Chem Sci. 2019 Oct 7;10(44):10428–35. doi: 10.1039/c9sc04158c (PMC6988741; doi:10.1039/c9sc04158c)
Supplement: Supplementary file 1 [file SC-010-C9SC04158C-s001.pdf]

Electronic Supplementary Material (ESI) for Chemical Science.
This journal is © The Royal Society of Chemistry 2019

Electronic Supplementary Information

Fabrication of Rigidity and Space Variable Protein Oligomers with Two Peptide Linker

	Page
Supporting Figures	S2-S19
Supporting Table	S20
Protein Sequences	S21
Experimental Methods	S23
References	S24

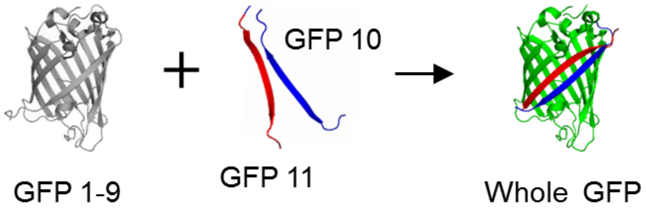

Fig. S1 Self-assembly of split superfolder GFP (sfGFP) with three fragments (GFP 1-9, GFP 10, and GFP 11). When GFP 10 and GFP 11 strands were brought together in close proximity, these two strands can self-assemble with GFP 1-9 to form a mature GFP protein with bright fluorescence.

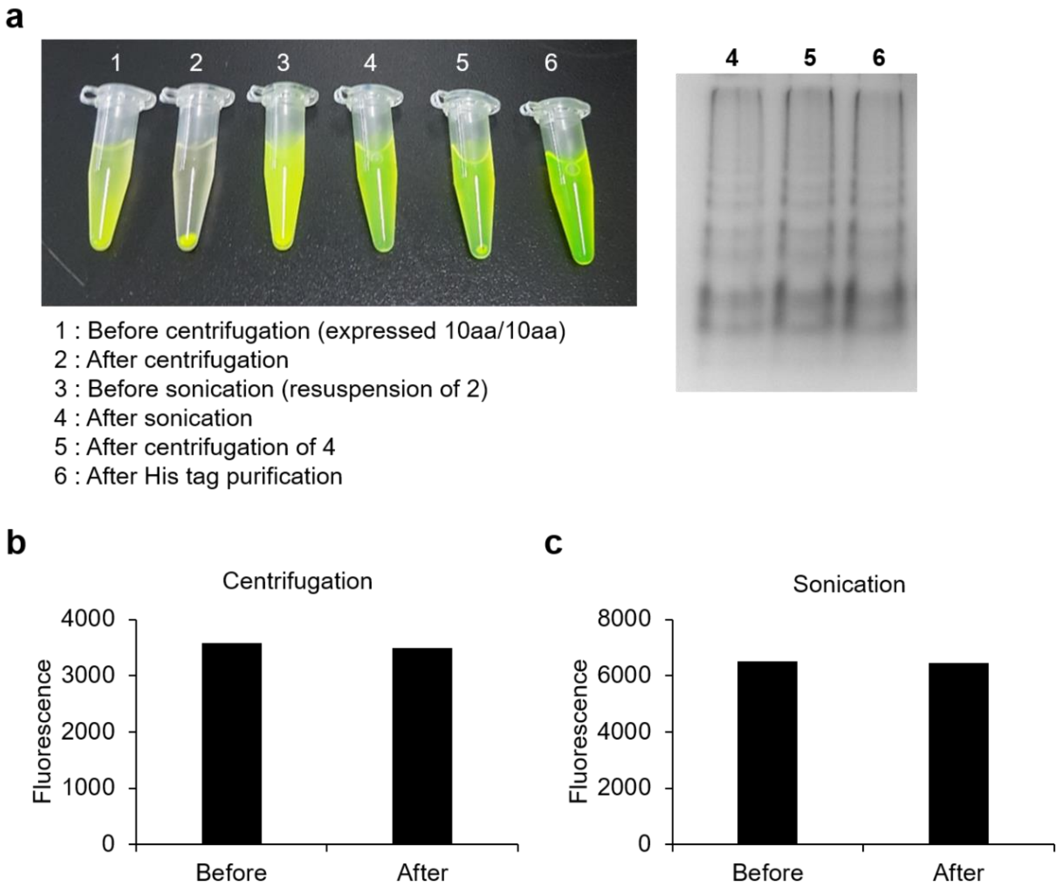

Fig. S2 Stability examination of GFP oligomers (10aa/10aa) during purification steps. (a) Solution images of GFP oligomer expressing cells and oligomer solutions during purification. A native gel image of cell lysates after sonication (4), centrifugation (5), and purified GFP oligomers (6) is shown right. (b) Fluorescence intensities of GFP oligomer expressing cells before and after centrifugation. (c) Fluorescence intensities of GFP oligomer expressing cells before and after sonication.

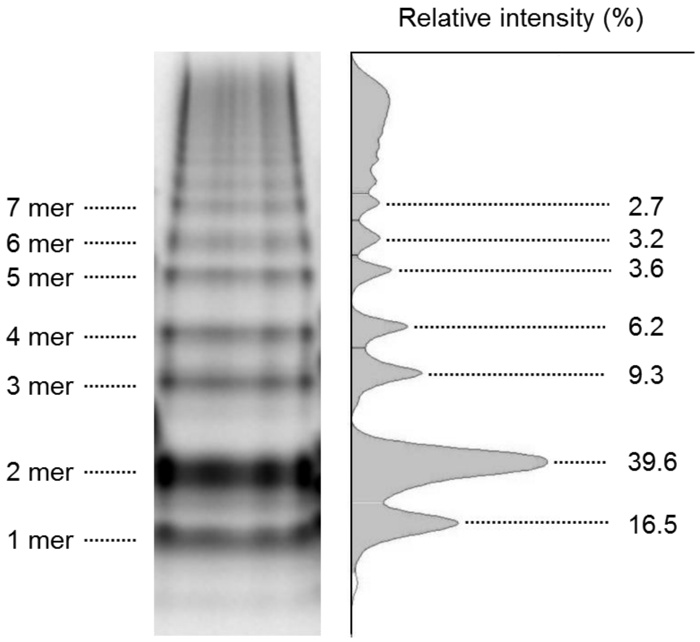

Fig. S3 Valency distributions of GFP oligomers. Relative fluorescence intensities of GFP oligomer (10aa/10aa) bands were obtained by Image 4.1 software.

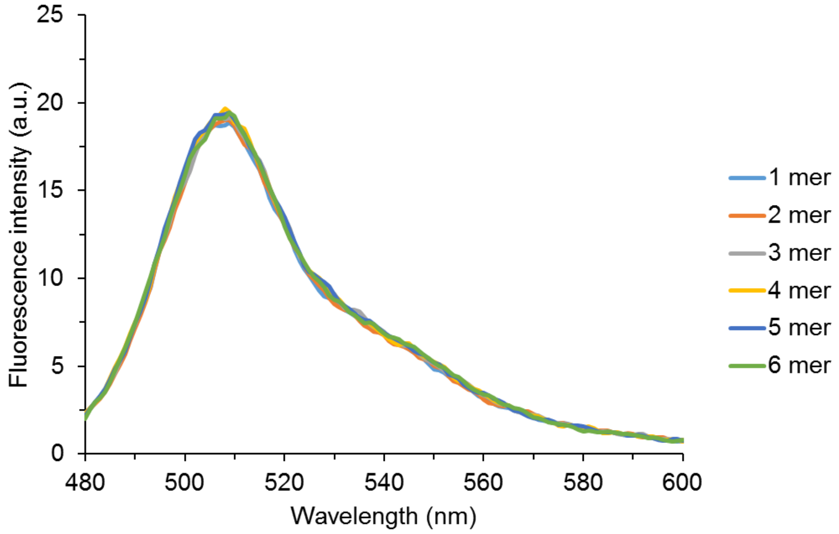

Fig. S4 Fluorescence emission spectra of gel-purified GFP oligomers (1 mg/mL) from monomer to hexamer.

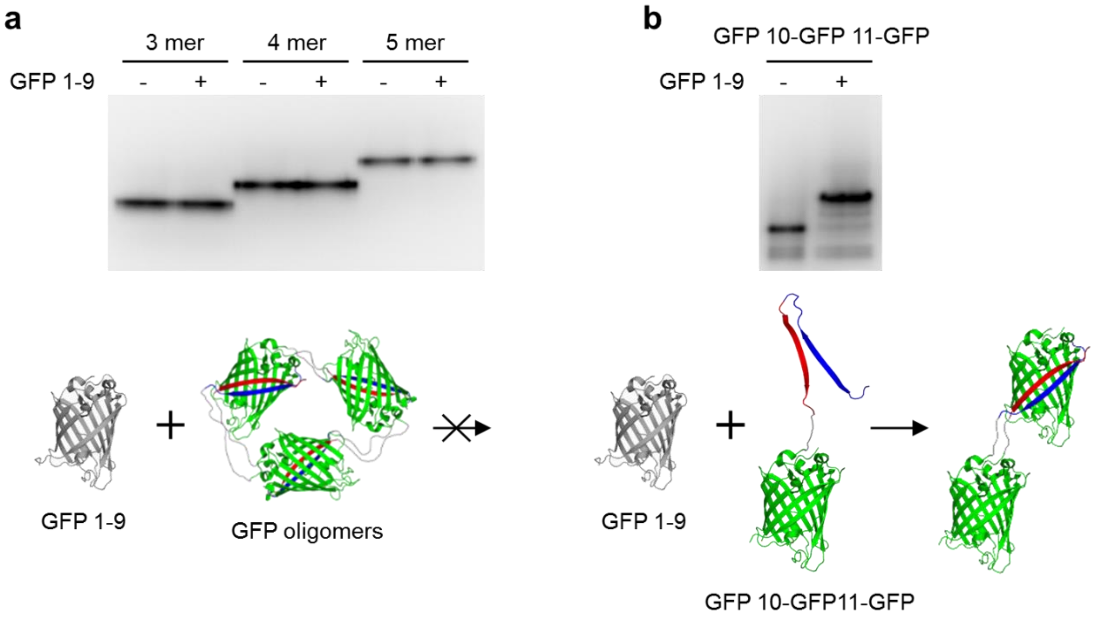

Fig. S5 Characterization of circularized GFP oligomers (a) *In vitro* reconstitutions of gel-purified GFP oligomers with the GFP 1-9 fragment. Trimer, tetramer, and pentamer of GFP oligomers (10aa/10aa) (5 μM) were incubated with two-fold excess GFP 1-9 (10 μM). No band shifts were observed. (b) Positive control of assembly between GFP 10-GFP 11-GFP (5 μM) and GFP 1-9 (10 μM). The gel analysis was conducted by fluorescent image analyzer. Schematics of assays are described below.

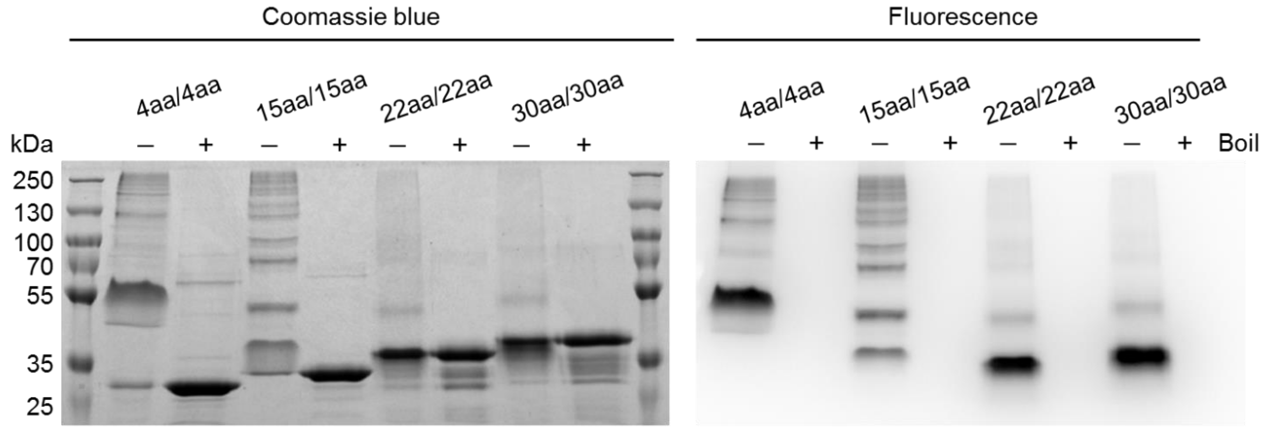

Fig. S6 SDS-PAGE analysis of GFP oligomers bearing length-varied flexible two peptide linkers with or without boiling.

a

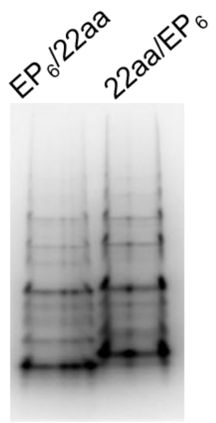

b

Name	Yield (250 mL culture)
EP_6/22aa	20.1 mg
22aa/EP_6	16.5 mg

Fig. S7 GFP oligomers with reversed two peptide linkers. (a) Native PAGE analysis of GFP oligomers with EP_6/22aa and its reversed linker variant 22aa/EP_6. (b) Expression yields of GFP oligomers.

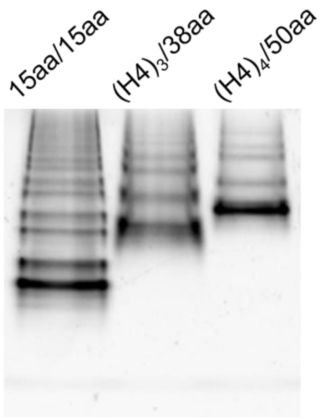

Linker sequence of $(H4)_4$/50aa

Linker 1:
GSGS$(EAAAK)_4$ALEA$(EAAAK)_4$GSGS$(EAAAK)_4$GSGS$(EAAAK)_4$
GSGS

Linker 2:
GGSEGGSGGTGSGGSGGGGGSGGGSTSGGGSEGSGGSGG
GSGGGGSGGGS

Fig. S8 Native PAGE analysis of GFP oligomers with 15aa/15aa, $(H4)_3$/38aa, and $(H4)_4$/50aa linkers. Linker sequences of $(H4)_4$/50aa are depicted below.

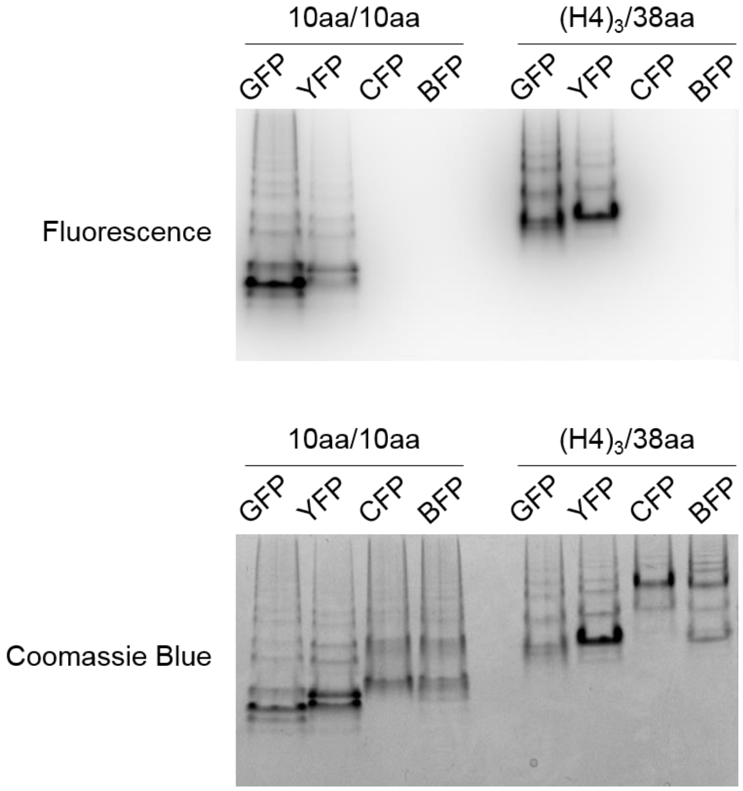

Fig. S9 Native PAGE analysis of GFP color variant oligomers with short (10aa/10aa) and long ($(H4)_3$/38aa) linkers. The gel was analyzed by fluorescent analyzer (top) and Coomassie blue staining (bottom). Excitation and emission filters for imaging cover all fluorescent proteins. Single mutations: YFP T203Y, CFP Y66F, and BFP Y66H.

Note: YFP oligomers were successfully formed, nearly similar to GFP, but with weaker fluorescence signals. Oligomeric assembly was clearly less efficient for CFP and BFP without any fluorescence signals. It is likely due to weakened complementarity of tripartite split FP fragments by mutations of color variants.

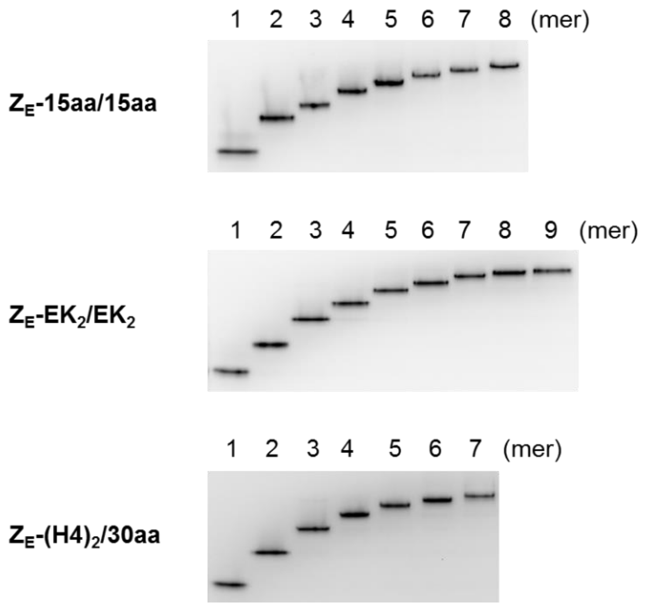

Fig. S10 Gel-purified Z$_E$ fused GFP oligomers with various flexible and rigid two peptide linkers.

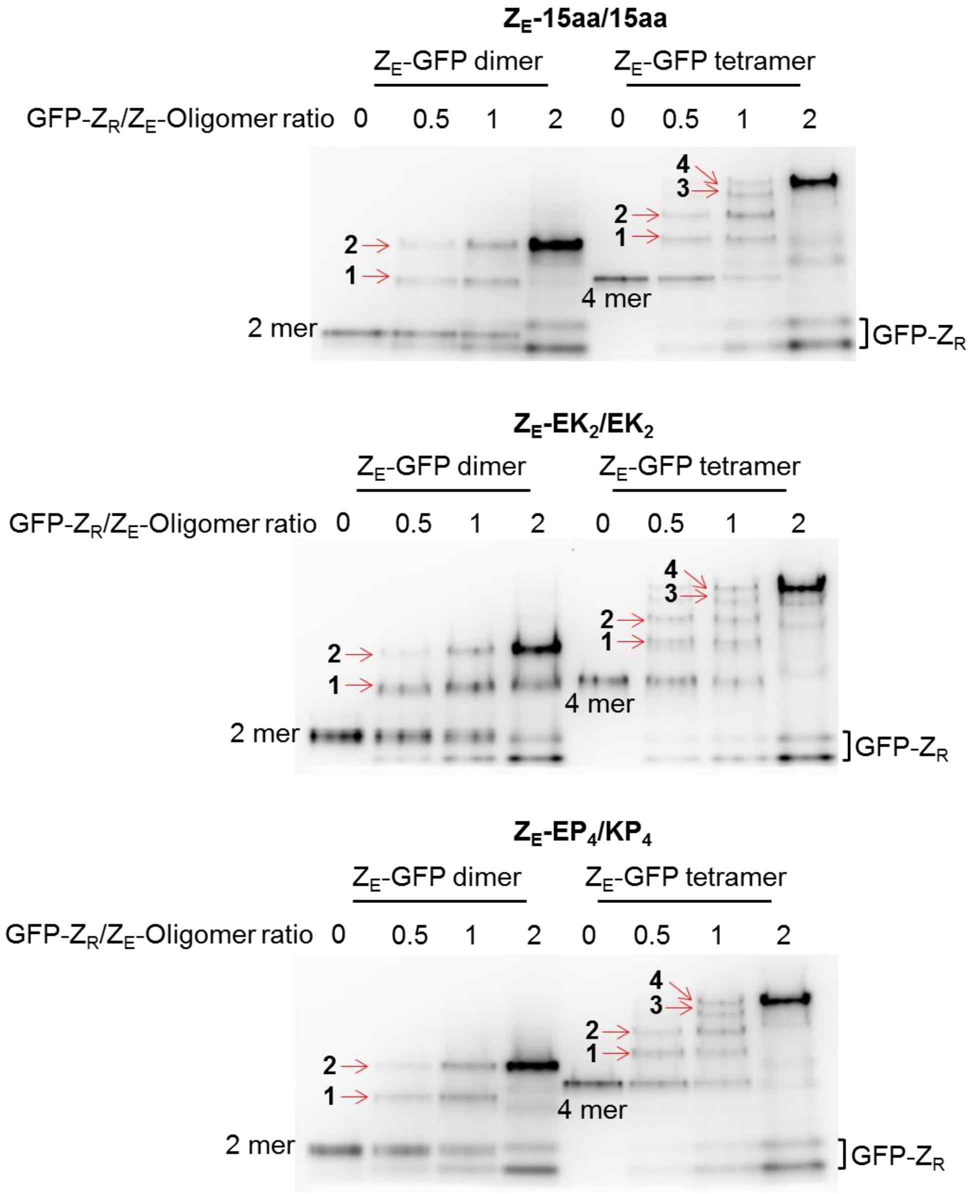

Fig. S11 Electrophoretic mobility shift assays of Z_E fused GFP dimers and tetramers binding to GFP-Z_R. Oligomers (1 μM Z_E concentration) were incubated with GFP-Z_R (0.5, 1, and 2 μM). Numbers of bound GFP-Z_R on Z_E-GFP dimer and tetramer are indicated on corresponding protein complex bands with red arrows.

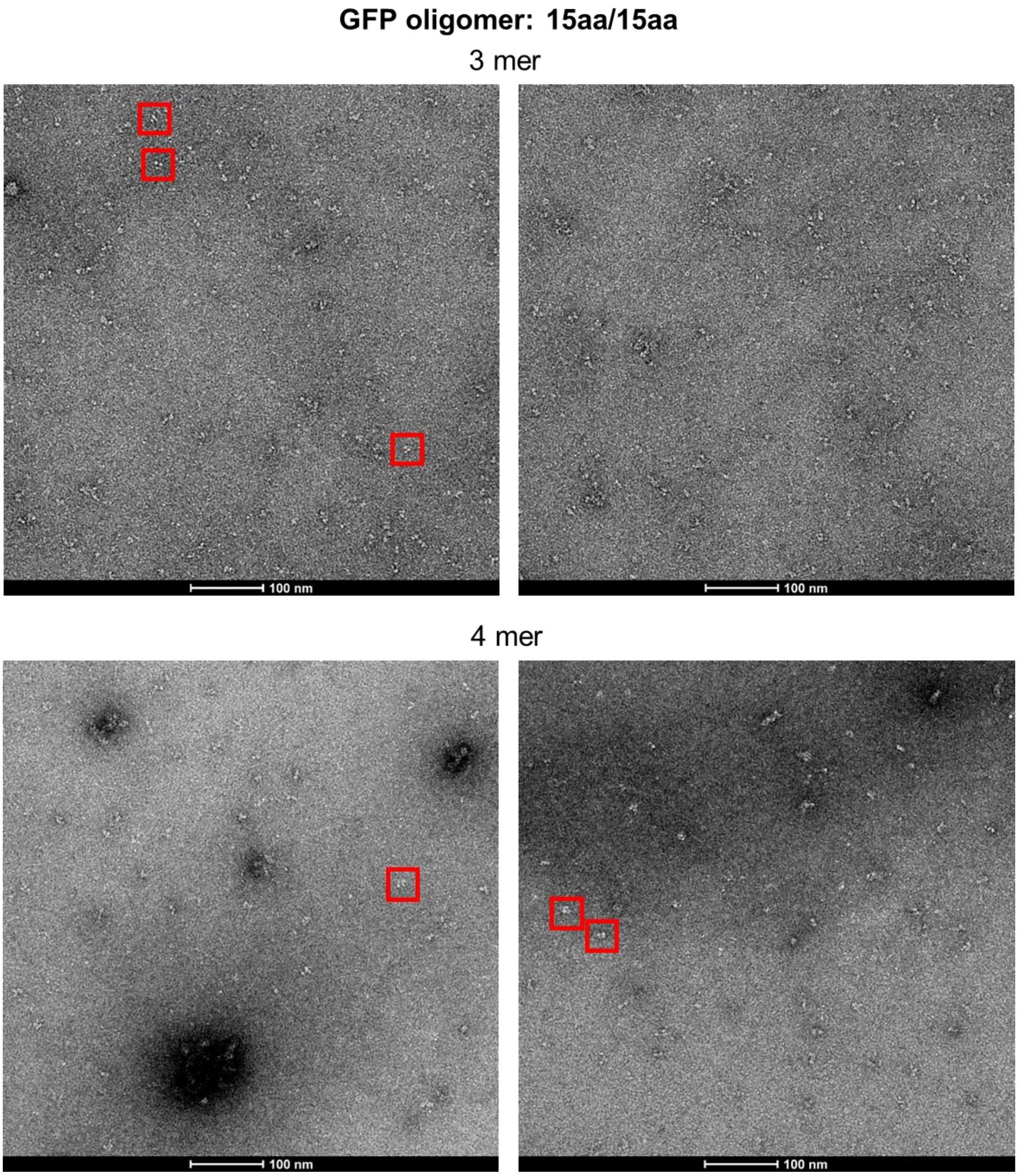

Fig. S12 TEM images of 15aa/15aa GFP trimer (top) and tetramer (bottom). Representative TEM images of individual GFP oligomers that are shown in Figure 6a are indicated with red boxes.

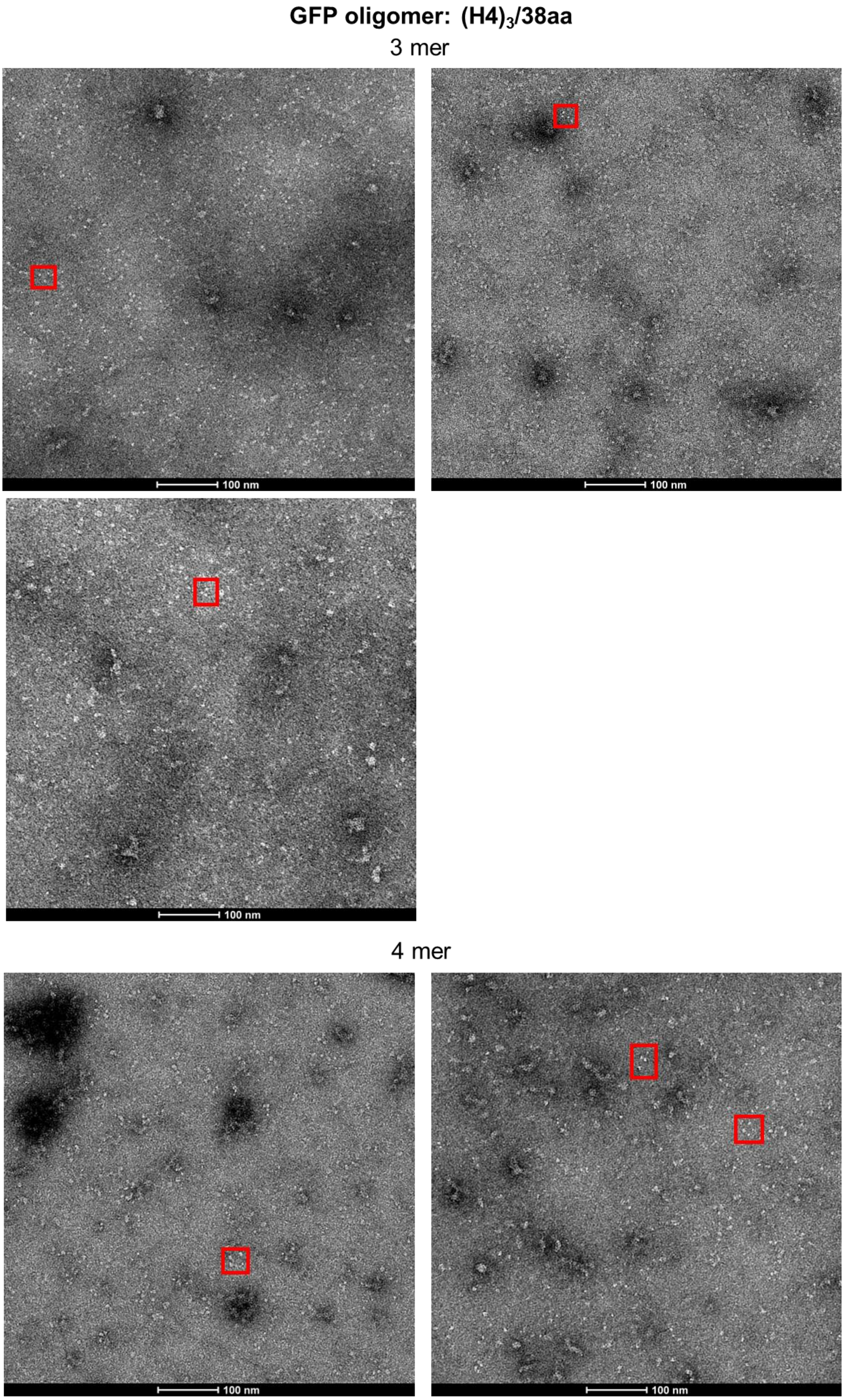

Fig. S13 TEM images of $(H4)_3$/38aa GFP trimer (top) and tetramer (bottom). Representative TEM images of individual GFP oligomers that are shown in Figure 6a are indicated with red boxes.

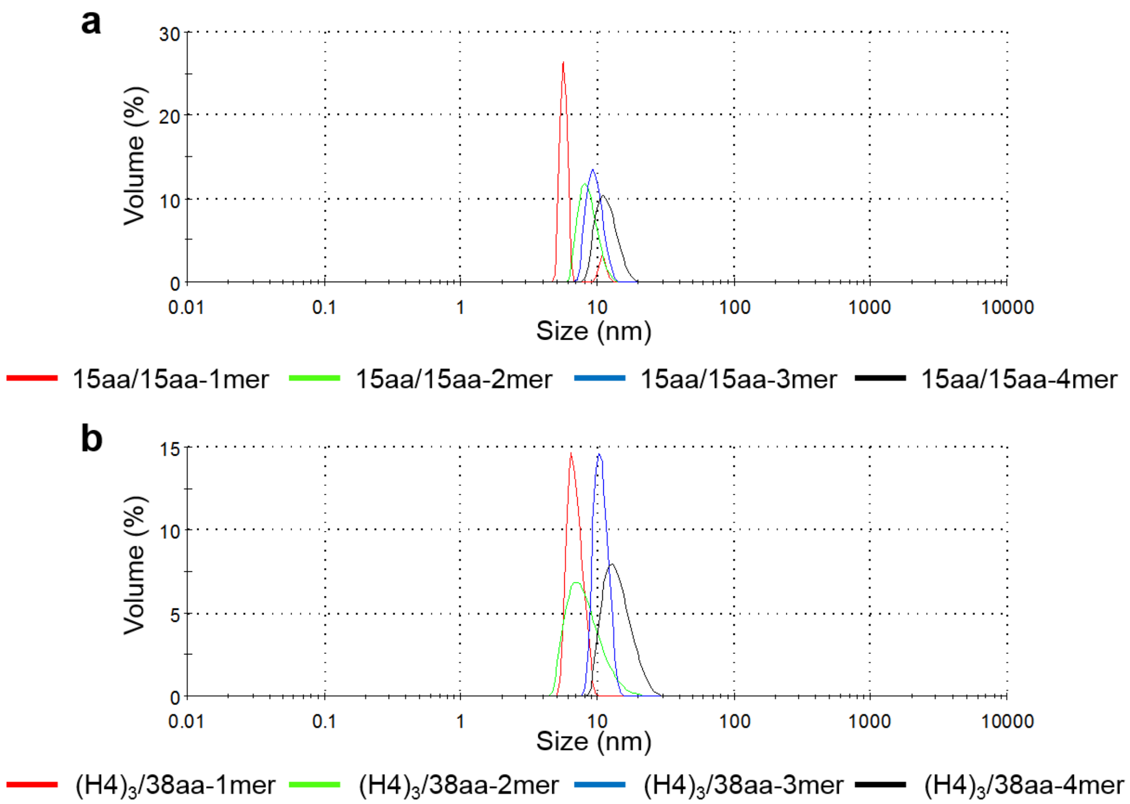

Protein	Size average (nm)	Standard Variation	Coefficient of Variation (%)
15aa/15aa-1mer	5.7	0.35	6.1
15aa/15aa-2mer	9.2	1.4	15.2
15aa/15aa-3mer	10.0	1.3	13.0
15aa/15aa-4mer	12.8	2.2	17.2
$(H4)_3$/38aa-1mer	7.2	0.9	12.5
$(H4)_3$/38aa-2mer	10.8	3.5	32.4
$(H4)_3$/38aa-3mer	11.1	1.3	11.7
$(H4)_3$/38aa-4mer	16.8	4.0	23.8

Fig. S14 Dynamic light scattering (DLS) analysis of (a) 15aa/15aa and (b) $(H4)_3$/38aa GFP oligomers. Mean diameters, standard variations and coefficient of variations are described on the table below.

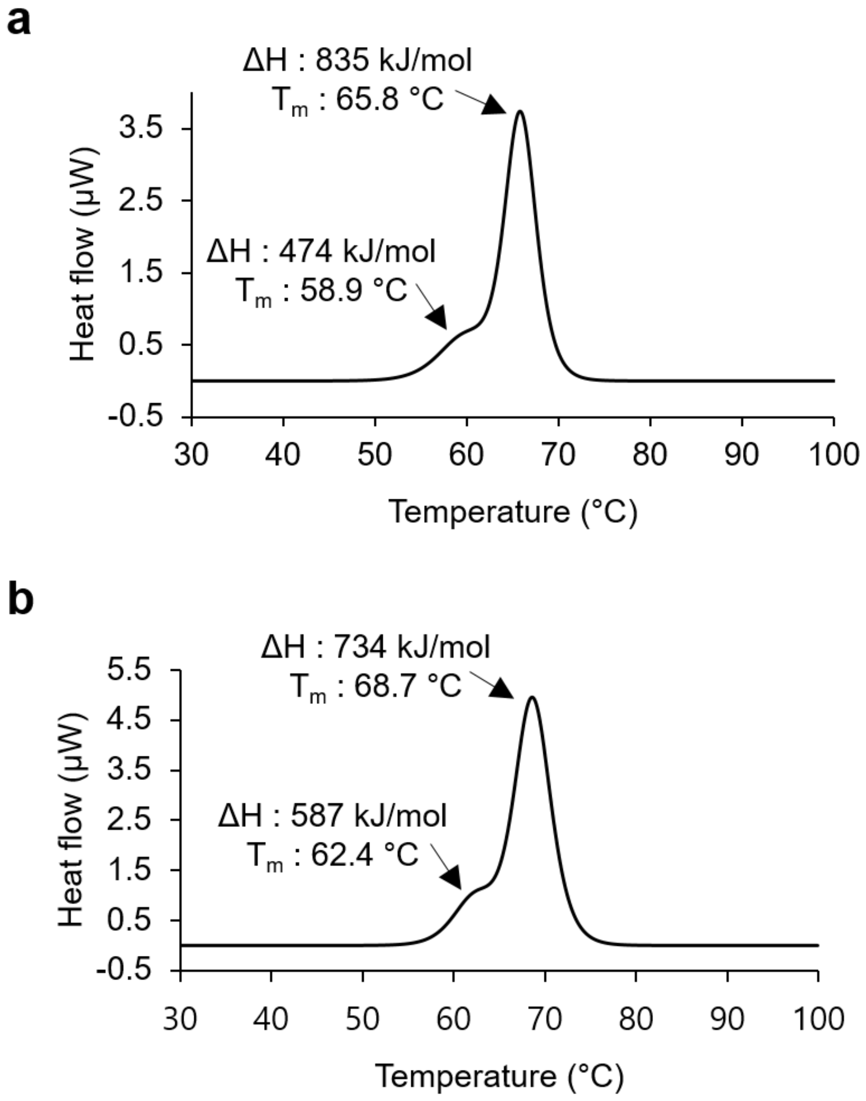

Fig. S15 Differential scanning calorimetry (DSC) analysis of GFP oligomers. The DSC spectra of (a) 15aa/15aa and (b) $(H4)_3/(H4)_3$ GFP oligomers are plotted with the heat flow as a function of temperature. Calculated melting temperatures (T_m) and corresponding enthalpy changes are depicted in the graph.

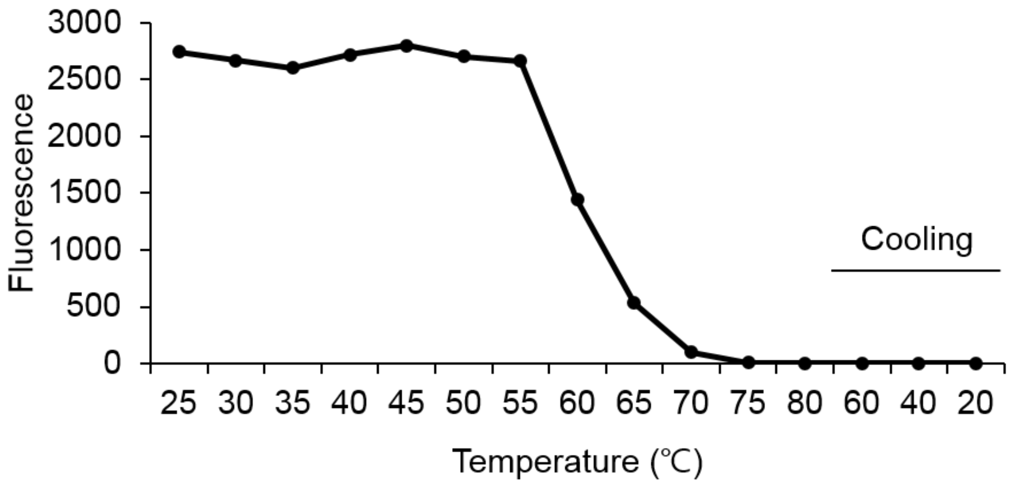

Fig. S16 Temperature stability of GFP oligomer (10aa/10aa). 10aa/10aa GFP oligomers were heated from 25 to 80 °C and then cooled down to 20 °C. Fluorescence intensities were measured after incubation for 30 min at each temperature.

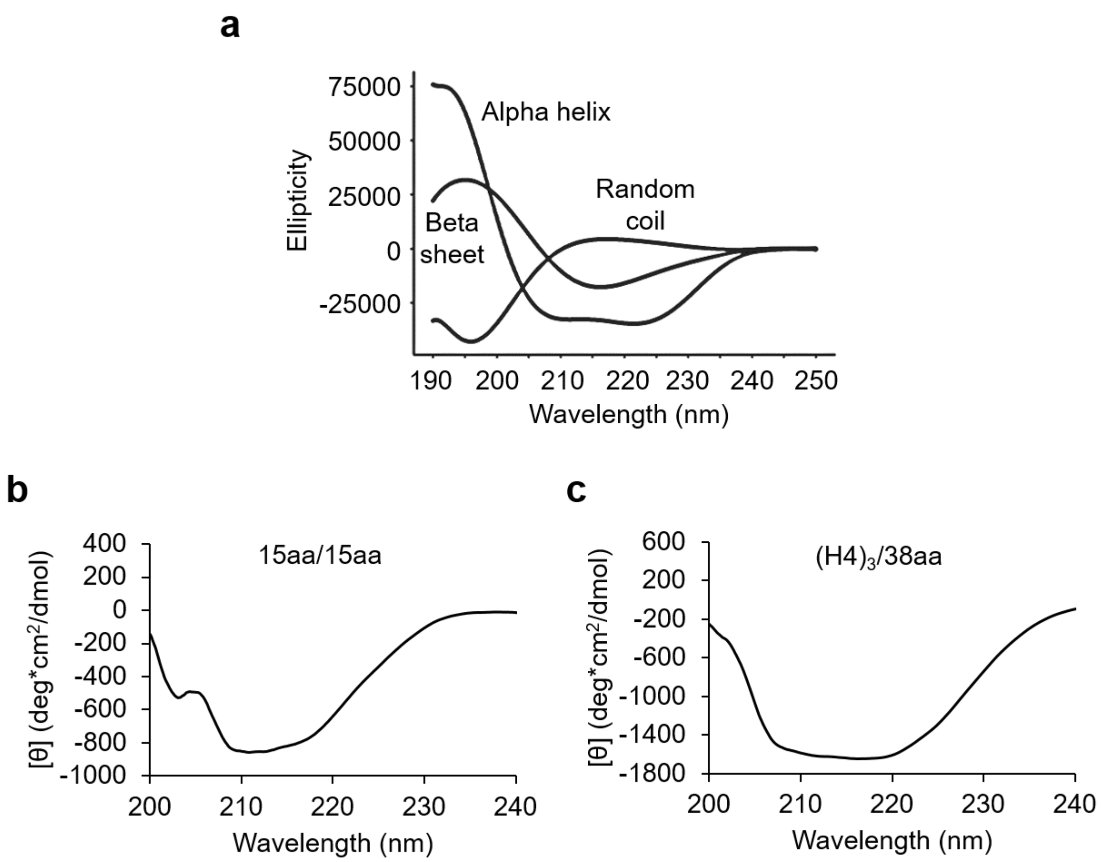

Fig. S17 Circular dichroism (CD) analysis. (a) Standard CD spectra of alpha-helix, beta-sheet, and random-coil. The CD spectra of (b) 15aa/15aa and (c) $(H4)_3$/38aa oligomers.

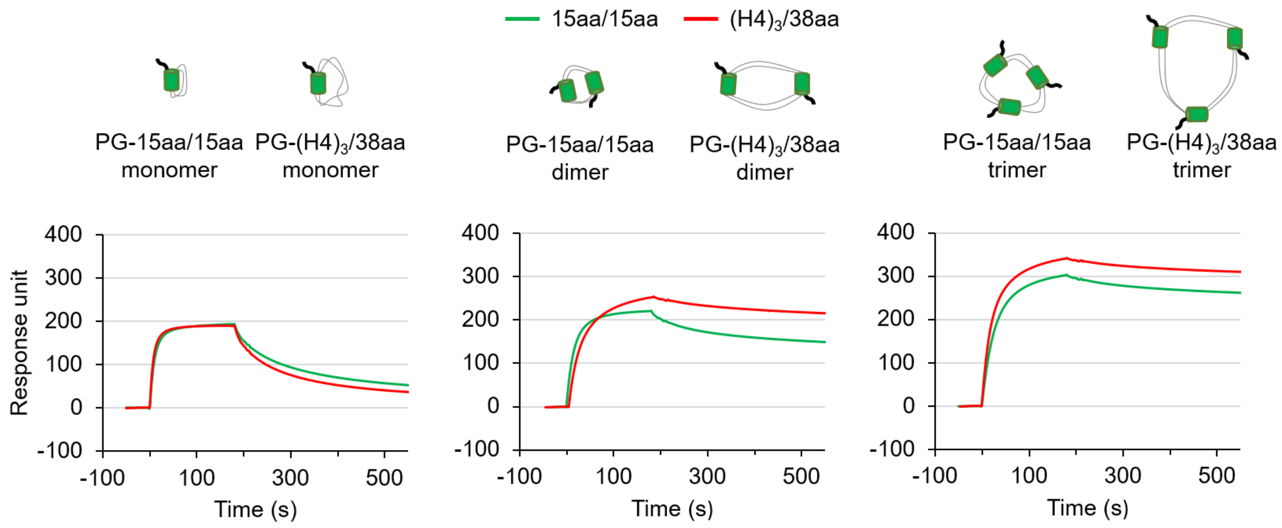

Fig. S18 Reconstructed SPR sensorgrams from Fig. 7b (PG-GFP oligomer binding to the high density Fc domain surface). SPR sensorgrams are organized to compare GFP oligomers with the same valency but with different linkers (green: 15aa/15aa, red: $(H4)_3$/38aa).

Supporting Table

Table S1. Artificial protein oligomers with varied linkers between protein building blocks.

Binding nature	Interaction pair	Form	Dispersity (valency)	Linker variation	References
Protein-peptide	S-peptide and S-protein	Circular	Polydisperse (1 to 4 mer)	Poly ethylene glycol (EG) (5, 18 EG)[a]	1
Enzyme-inhibitor	$DHFR_2$ and bisMTX	Mostly circular	Polydisperse (1 to 8 mer)	Peptide (Amino acids, aa) (1, 3, 7, 13 aa)[b]	2
Enzyme-inhibitor & Homodimer (enzyme)	a) $DHFR_2$ and bisMTX b) hHint1	Mostly circular	Polydisperse (1 to >6 mer)	Peptide (1, 3, 7, 15 aa)[c]	3
Protein-cofactor	Cytochrome b_{562} and heme	Mostly linear	Polydisperse (up to >100 mer)	Ethylene glycol (0, 2, 3 EG)	4
Protein-peptide	Split superfolder GFP	Circular	Polydisperse; Discrete isolation (1 to 10 mer)	Peptide (10 to 76 aa)	This study

[a] When the PEG linker with 18 EG units was added between protein building blocks, mostly 1 mer and 2 mer were fabricated.

[b] With the 13 aa peptide linker, only up to 5 mer of protein oligomers were fabricated.

[c] Monomer units contain two proteins ($DHFR_2$ and hHint1), which are linked by indicated peptide linkers. With the 15 aa peptide linker, oligomers only between 1 mer to 3 mer were dominantly fabricated.

Protein Sequences

I. GFP oligomers with two peptide linkers

GFP oligomer monomeric subunit (10aa/10aa)

MYTMDLPDDHYLSTQTILSKDLNGTDVGSGGGSRKGEELFTGVVPILIELDGDVNGHKFFVRGEGEGDATIGKL
SLKFICTTGKLPVPWPTLVTTLTYGVQCFSRYPDHMKRHDFFKSAMPEGYVQERTIYFKDDGTYKTRAEVKFEG
DTLVNRIELKGIDFKEDGNILGHKLEYNFNSHKVYITADKQNNGIKANFTIRHNVEDGSVQLADHYQQNTPIGDG
PVLLPGGGSGGGSTSEKRDHMVLLEYVTAAGITDAS

Blue: GFP 10, purple: GFP 11, green: GFP 1-9, underline: linkers. For other linker variants, linker sequences are summarized in Table S1.

II. Functional peptide and proteins

All functional peptide and proteins were fused to the C-terminus of GFP monomers via a peptide linker (GGGGS).

Leucine zipper, Z_E peptide

LEIEAAALEQENTALETEVAELEQEVQRLENIVSQYRTRYGPL

SpyCatcher

GAMVDTLSGLSSEQGQSGDMTIEEDSATHIKFSKRDEDGKELAGATMELRDSSGKTISTW
ISDGQVKDFYLYPGKYTFVETAAPDGYEVATAITFTVNEQGQVTVNGKATKGDAHI

Protein G

TYKLVINGKTLKGETTTKAVDAETAEKAFKQYANDNGVDGVWTYDDATKTFTVTE

mCherry

MVSKGEEDNMAIIKEFMRFKVHMEGSVNGHEFEIEGEGEGRPYEGTQTAKLKVTKGGPLPFAWDILSPQFMYGS

KAYVKHPADIPDYLKLSFPEGFKWERVMNFEDGGVVTVTQDSSLQDGEFIYKVKLRGTNFPSDGPVMQKKTMG
WEASSERMYPEDGALKGEIKQRLKLKDGGHYDAEVKTTYKAKKPVQLPGAYNVNIKLDITSHNEDYTIVEQYE
RAEGRHSTGGMDELYK

III. Leucine zipper tagged GFP

GFP-Z_R

MKGEELFTGVVPILVELDGDVNGHEFSVRGEGEGDATIGKLTLKFICTTGKLPVPWPTLVTTLTYGVQCFSRYPD
HMKRHDFFKSAMPEGYVQERTISFKDDGKYKTRAVVKFEGDTLVNRIELKGTDFKEDGNILGHKLEYNFNSHD
VYITADKQENGIKAEFTVRHNVEDGSVQLADHYQQNTPIGDGPVLLPDNHYLSTQTVLSKDPNEKRDHMVLHE
YVNAAGITGGGSGGGTGGGSGGGLEIRAAALRRRNTALRTRVAELRQRVQRLRNEVSQYETRYGPL

Blue: Z_R peptide, green: GFP, underline: linker.

Experimental Methods

Expression and purification of GFP oligomers. All DNA sequences were amplified by PCR using synthetic oligonucleotide primers. The resulting genes were ligated into the pET-28a (Novagen) or pET21a (Novagen) expression vector. The cloned plasmid vectors were transformed to E. coli BL21(DE3) competent cells for protein expression. Protein expression was induced with 1 mM IPTG at an OD600 of 0.7. Expression was allowed to continue for 16 h at 20 °C. Cells were harvested by centrifugation, and the pellets were washed with PBS buffer. After washing with His-binding-buffer (500 mM NaCl, 50 mM Tris-Cl, pH 8.0), cells were lysed by sonication. His6-tagged proteins were purified from soluble protein fractions of cell lysates using a Ni-NTA (Qiagen) column. Oligomer concentrations were measured by Bradford assays and fluorescence images of oligomers were obtained by Chemi-doc (Biorad).

Gel purification of discrete GFP oligomers. GFP oligomer mixtures were separated on an 8% native PAGE gel at 250 V for 200 min. Each fluorescent oligomer band was sliced under ultraviolet light. Excised gels were transferred into a dialysis membrane (Spectrum Labs, MWCO 3,500) and filled up with the gel running buffer (192 mM glycine and 25 mM Tris base) at 4 °C. GFP oligomers were then eluted from the gels in a horizontal gel electrophoresis apparatus by running at 100 V for 90 min at 4 °C with a pre-chilled running buffer. The eluted fractions were collected, and NaCl was added to a final concentration of 125 mM. Proteins were concentrated by polyethylene glycol 8,000 (LPS solution) if needed. After additional dialysis into PBS, GFP oligomers were stored at 4 °C.

Circular dichroism (CD) spectroscopy. Circular dichroism spectra were obtained on a J-1500 CD spectropolarimeter (JASCO) using 0.5 mg/mL of samples in buffer containing 20 mM NaH_2PO_4 at pH 7.4. CD spectra were measured between 200 nm and 240 nm.

Differential scanning calorimetry (DSC) analysis. DSC profiles of GFP oligomers were obtained by using a Nano DSC (TA Instruments). Each sample (2 mg/mL) was dialyzed against a PBS buffer and degassed before measurement. The PBS buffer was used as a reference sample. All experiments were performed between 0 °C and 100 °C with a scanning rata of 0.3 K/min. The melting temperature (T_m) was acquired by simulation using a two state scaled model.

Electrophoretic mobility shift assay (EMSA). Electrophoretic mobility shift assays were conducted to determine the valencies of GFP oligomers. GFP oligomers bearing C-terminal ZE peptides (1 μM) were mixed with varying concentrations of GFP-Z_R (0.5 - 2 μM). The mixtures were incubated for 1

h at room temperature. After incubation, mixtures were electrophoresed and protein bands were monitored by following fluorescence signals of GFP.

Size-exclusion chromatography. GFP oligomers were prepared in an elution buffer (150 mM NaCl, 50 mM Tris pH 7.2) and injected onto a Superdex 200 column (10/300 GL, GE Healthcare). Chromatograpy was conducted at a flow rate of 0.2 mL/min with an elution buffer, and protein elution was monitored with a UV detector at 280 nm.

Negative-stain transmission electron microscopy (TEM). GFP oligomers were adsorbed to carbon grids and negatively stained with 0.75% uranyl formate for 2 min. TEM images were acquired with a 4K×4K Eagle HS CCD (charge-coupled device) camera (FEI) on a Tecnai T120 microscope (FEI) at 120 kV. Images were taken at a magnification of × 67,000 and defocus settings ranging from -1.4 to -1 mm.

Surface plasmon resonance (SPR) analysis. SPR experiments were carried out on a Biacore T200 instrument (GE Healthcare) using a HEPES buffer (pH 7.4) as a running solution. Human IgG-Fc fragments (Bethyl Laboratories) were immobilized onto a gold sensor chip, which was SAM modified using $HSC_{11}EG_3OH$ and $HSC_{11}EG_3COOH$ (Prochimia) at a 9 to 1 ratio, using EDC/NHS amine coupling to approximately 70 RU for a low density or 700 RU for a high density surface. Remaining reactive groups were blocked with 1 M ethanolamine. All samples were diluted in a HEPES buffer and 100 nM of protein G-fused GFP oligomers were injected to a human IgG-Fc fragments bound surface at a flow rate of 30 μL/min. Association and dissociation of protein G-fused GFP oligomers were observed for 180 s and 320 s, respectively. After each experiment, chips were regenerated by injecting 20 μL of 10 mM NaOH. The reference surface was subtracted to normalize refractive index differences.

References

1 M. M. C. Bastings, T. F. A. de Greef, J. L. J. van Dongen, M. Merkx and E. W. Meijer, *Chemical Science*, 2010, **1**, 79-88.

2 J. C. Carlson, S. S. Jena, M. Flenniken, T. F. Chou, R. A. Siegel and C. R. Wagner, *J. Am. Chem. Soc.*, 2006, **128**, 7630-7638.

3 T.-F. Chou, C. So, B. R. White, J. C. T. Carlson, M. Sarikaya and C. R. Wagner, *ACS Nano*, 2008, **2**, 2519-2525.

4 H. Kitagishi, K. Oohora, H. Yamaguchi, H. Sato, T. Matsuo, A. Harada and T. Hayashi, *J. Am. Chem. Soc.*, 2007, **129**, 10326-10327.
